# Supplementary material for: Self-medication practices to prevent or manage COVID-19: A systematic review
Source: PLoS One. 2021 Nov 2;16(11):e0259317. doi: 10.1371/journal.pone.0259317 (PMC8562851; doi:10.1371/journal.pone.0259317)
Supplement: S1 Table — (DOCX) [file pone.0259317.s002.docx]

# S1 Table. Search strategies.

**Scopus**

| ***Step*** | ***Search query*** | ***Results*** |
| --- | --- | --- |
| #1 | TITLE-ABS-KEY ( "2019-nCoV" OR "COVID-19" OR "coronavirus disease 2019" OR "SARS-CoV-2" OR "HCoV-2019" OR "hcov" OR "NCOVID-19" OR "severe acute respiratory syndrome coronavirus 2" OR "severe acute respiratory syndrome corona virus 2" OR "SARS-CoV2" OR covid2019 OR "COVID-19" OR covid19 OR 2019ncov OR "2019 ncov" OR "novel coronaviru*" OR "novel coronaviruses" OR "novel corona virus" OR "novel corona*" OR covid19 OR "covid 19" OR "sars cov 2" OR sars2 OR "new corona*" ) OR ( TITLE-ABS-KEY ( coronaviru* OR "corona viru*" OR "pneumonia viru*" OR cov OR ncov ) W/4 ( wuhan OR china OR novel ) ) | 110,848 |
| #2 | TITLE-ABS-KEY ("self medication" OR "nonprescription drug*" OR "non prescription drug*" OR "over-the-counter drug*" OR "OTC drug*" OR "drug hoarding" OR "drug utilization" OR "medication utilization" OR "pre-hospitalary medication") | 60,412 |
| #3 | #1 AND #2 | 135 |

**Pubmed/Medline**

| ***Step*** | ***Search query*** | ***Results*** |
| --- | --- | --- |
| #1 | (("COVID-19"[Mesh] OR "COVID-19"[Supplementary Concept] OR "severe acute respiratory syndrome coronavirus 2"[Supplementary Concept] OR "severe acute respiratory syndrome coronavirus 2"[tiab] OR "2019nCoV"[tiab] OR "2019-nCoV"[tiab] OR "novel coronavirus"[tiab] OR "novel coronaviruses"[tiab] OR "novel corona virus"[tiab] OR "COVID19"[tiab] OR "COVID-19"[tiab] OR "SARS-CoV-2"[tiab] OR "SARS-CoV2"[tiab] OR "sars2"[tiab] OR "new coronavirus"[tiab] OR "new coronaviruses"[tiab] OR "ncov 2019"[tiab] OR "sars coronavirus 2"[tiab]) OR (("coronavirus"[tiab] OR "coronavirus's"[tiab] OR "coronaviruscpe"[tiab] OR "coronaviruse"[tiab] OR "coronaviruses"[tiab] OR "coronaviruses"[tiab] OR "coronaviruslike"[tiab] OR "corona virus"[tiab] OR "corona viruses"[tiab] OR "pneumonia virus"[tiab] OR "pneumonia viruses"[tiab] OR "cov"[tiab] OR "ncov"[tiab]) AND ("outbreak"[tiab] OR "WUHAN"[tiab] OR "2019"[tiab])) OR ("new"[All Fields] AND ("corona virus"[tiab] OR "corona viruses"[tiab]))) NOT "middle east"[Title] | 97,782 |
| #2 | ("Self Medication"[MeSH] OR "Self Medication"[tiab] OR "Self Medication"[tw] OR "Nonprescription Drugs"[MeSH] OR "Nonprescription Drugs"[tiab] OR "Nonprescription Drugs"[tw] OR "Non prescription Drugs"[tiab] OR "Non prescription Drugs"[tw] OR "over-the-counter drugs"[tiab] OR "over-the-counter drugs"[tw] OR "OTC drugs"[tiab] OR "OTC drugs"[tw] OR "drug hoarding"[tiab] OR "drug hoarding"[tw] OR "drug utilization"[tiab] OR "drug utilization"[tw] OR "medication utilization"[tiab] OR "medication utilization"[tw]) OR (medicine*[TI] AND use[TI]) | 45,070 |
| #3 | #1 AND #2 | 92 |

**Embase**

| ***Step*** | ***Search query*** | ***Results*** |
| --- | --- | --- |
| #1 | coronavir* AND [2020-2021]/py | 96,925 |
| #2 | 'coronavirus disease 2019'/exp OR 'coronavirus disease 2019' | 84,420 |
| #3 | '2019 novel coronavirus'/exp OR '2019 novel coronavirus' | 22,215 |
| #4 | '2019-ncov' | 1,493 |
| #5 | 'wuhan coronavirus' | 31 |
| #6 | '2019 new coronavirus' OR '2019 novel coronavirus' | 1,292 |
| #7 | ('coronaviridae'/exp OR coronaviridae) AND wuhan:ti,ab,kw | 2,198 |
| #8 | 'severe acute respiratory syndrome coronavirus 2' OR 'covid-19' OR 'covid19' OR '2019-ncov' OR 'sars-cov-2' OR 'sars-cov2' OR '2019 novel coronavirus infection' OR 'coronavirus disease 2019' OR 'coronavirus disease-19' OR '2019 novel coronavirus disease' OR ('pneumonia' AND 'wuhan' AND '2019') | 98,543 |
| #9 | ('sars coronavirus'/exp OR 'sars coronavirus' OR (('covid'/exp OR covid) AND 19:ab,ti) OR (severe AND acute AND respiratory AND ('syndrome'/exp OR syndrome) AND ('coronavirus'/exp OR coronavirus) AND 2:ab,ti) OR '2019 ncov':ab,ti OR 2019ncov:ab,ti OR 'covid 19':ab,ti OR 'sars cov 2':ab,ti OR (('wuhan'/exp OR wuhan) AND coronavirus:ab,ti) OR (('wuhan'/exp OR wuhan) AND ('pneumonia'/exp OR pneumonia) AND virus:ab,ti) OR (('covid19'/exp OR covid19) AND virus:ab,ti) OR (('covid 19'/exp OR 'covid 19') AND virus:ab,ti) OR 'coronavirus disease 2019':ab,ti OR (('virus'/exp OR virus) AND 'sars cov 2':ab,ti) OR (sars2 AND '2019 ncov':ab,ti) OR '2019 novel coronavirus':ab,ti) AND [2020-2021]/py | 89,424 |
| #10 | #1 OR #2 OR #3 OR #4 OR #5 OR #6 OR #7 OR #8 OR #9 | 101,072 |
| #11 | (2020:py OR 2021:py) | 1,940,937 |
| #12 | #10 AND #11 | 100,888 |
| #13 | 'non prescription drug'/exp OR 'non prescription drug' | 14,337 |
| #14 | 'self medication':ti,ab,kw OR 'non prescription drug*':ti,ab,kw OR 'nonprescription drug*':ti,ab,kw OR 'over-the-counter drug*':ti,ab,kw OR 'OTC drug*':ti,ab,kw OR 'drug hoarding':ti,ab,kw OR 'drug utilization':ti,ab,kw OR 'medication utilization':ti,ab,kw OR 'pre-hospitalary medication':ti,ab,kw | 15,712 |
| #15 | 'self medication'/exp OR 'self medication' | 12,885 |
| #16 | #13 OR #14 OR #15 | 33,732 |
| #17 | #12 AND #16 | 129 |

**Web of Science**

| ***Step*** | ***Search query*** | ***Results*** |
| --- | --- | --- |
| #1 | TS= ( "2019-nCoV" OR "COVID-19" OR "SARS-CoV-2" OR "HCoV-2019" OR "hcov" OR "NCOVID-19" OR "severe acute respiratory syndrome coronavirus 2" OR "severe acute respiratory syndrome corona virus 2" OR "SARS-CoV2" OR covid2019 OR "COVID-19" OR covid19 OR 2019ncov OR "2019 ncov" OR "novel coronaviru*" OR "novel coronaviruses" OR "novel corona virus" OR "novel corona*" OR covid19 OR "covid 19" OR "sars cov 2" OR sars2 OR "new corona*" OR “new coronavirus” OR “coronavirus disease 2019” ) OR TS= ( ( coronaviru* OR "corona viru*" OR "pneumonia viru*" OR cov OR ncov ) NEAR/4 ( wuhan OR china OR novel ) ) | 120,283 |
| #2 | TS=("self medication" OR "nonprescription drug*" OR "non prescription drug*" OR "over-the-counter drug*" OR "OTC drug*" OR "drug hoarding" OR "drug utilization" OR "medication utilization" OR "pre-hospitalary medication" ) | 44,256 |
| #3 | #1 AND #2 | 74 |

**MedRxiv**

Go to <https://www.medrxiv.org/>

Click on “COVID-19 SARS-CoV-2 preprints from medRxiv and bioRxiv”

Click on “About”

Search:

| ***N°*** | ***Search terms*** | ***Results*** |
| --- | --- | --- |
| 1 | self-medication | 51 |
| 2 | nonprescription drug | 21 |
| 3 | over-the-counter drug | 62 |
|  | TOTAL | 134 |

**Scielo preprints**

Go to <https://preprints.scielo.org/index.php/scielo/preprints>

Search:

| ***N°*** | ***Search terms*** | ***Results*** |
| --- | --- | --- |
| 1 | self medication | 1 |

**Google Scholar**

Go to <https://scholar.google.com/>

**Search the following:** self medication medicine use covid

Review the first 100 results

**Google**

Go to <https://www.google.com.pe/>

**Search the following:** self medication medicine use covid

Review the first 100 results
