# Supplementary material for: Self-medication practices to prevent or manage COVID-19: A systematic review
Source: PLoS One. 2021 Nov 2;16(11):e0259317. doi: 10.1371/journal.pone.0259317 (PMC8562851; doi:10.1371/journal.pone.0259317)
Supplement: S2 Table — (DOCX) [file pone.0259317.s003.docx]

**S2 Table. List of articles excluded after full-text review.**

| **N°** | **Author** | **Year** | **Title** | **Final Decision** | **Reasons for exclusion** |
| --- | --- | --- | --- | --- | --- |
| 1 | Aliu P | 2020 | Increasing Use of Compassionate Use/Managed Access Channels to Obtain Medicines for Use in COVID-19 | Excluded | Did not show original results |
| 2 | Alpern JD | 2020 | Off-Label Therapies for COVID-19—Are We All In This Together? | Excluded | Did not show original results |
| 3 | Beyzarov E | 2020 | Global Safety Database Summary of COVID-19-Related Drug Utilization-Safety Surveillance: A Sponsor's Perspective | Excluded | Did not show original results |
| 4 | Cardoso CRB | 2021 | What happens in brazil? A pandemic of misinformation that culminates in an endless disease burden | Excluded | Did not show original results |
| 5 | Dalmat YM | 2020 | Over the counter chloroquine: Danger! | Excluded | Did not show original results |
| 6 | Gray PE | 2020 | The use of Traditional Chinese Medicines to treat SARS-CoV-2 may cause more harm than good | Excluded | Did not show original results |
| 7 | Malik M | 2020 | Self-medication during Covid-19 pandemic: challenges and opportunities | Excluded | Did not show original results |
| 8 | Mallhi TH | 2020 | Drug repurposing for COVID-19: a potential threat of self-medication and controlling measures | Excluded | Did not show original results |
| 9 | Tejada SF | 2020 | Self-medication promoted by the media: A hazard with consequences in the time of the covid-19 public health crisis | Excluded | Did not show original results |
| 10 | Wong A | 2020 | COVID-19 and toxicity from potential treatments: Panacea or poison | Excluded | Did not show original results |
| 11 | Molento MB | 2020 | COVID-19 and the rush for self-medication and self-dosing with ivermectin: A word of caution | Excluded | Did not show original results |
| 12 | Sachdeva M | 2020 | Risks of hydroxychloroquine use for COVID-19 prophylaxis | Excluded | Did not show original results |
| 13 | Silveira D | 2020 | COVID-19: Is There Evidence for the Use of Herbal Medicines as Adjuvant Symptomatic Therapy? | Excluded | Did not show original results |
| 14 | Tuccori M | 2020 | The Impact of the COVID-19 "Infodemic" on Drug-Utilization Behaviors: Implications for Pharmacovigilance | Excluded | Did not show original results |
| 15 | Huang ST | 2020 | Principles and treatment strategies for the use of Chinese herbal medicine in patients at different stages of coronavirus infection | Excluded | Did not show original results |
| 16 | Ientile G | 2020 | Covid-19 what community pharmacies are doing in the hardest-hit states | Excluded | Did not show original results |
| 17 | Varon A | 2020 | Traditional chinese medicine and COVID-19: should emergency practitioners use it? | Excluded | Did not show original results |
| 18 | Varrassi G | 2020 | Warning Against the Use of Anti-Inflammatory Medicines to Cure COVID-19: Building Castles in the Air | Excluded | Did not show original results |
| 19 | Calderón CA | 2020 | The Observatory of Self-Medication Behavior of the Universidad del Rosario and its role in the COVID-19 pandemic | Excluded | Did not show original results |
| 20 | Faqihi AHMA | 2020 | Self-medication practice with analgesics (NSAIDs and acetaminophen), and antibiotics among nursing undergraduates in University College Farasan Campus, Jazan University, KSA | Excluded | Was not performed in the context of COVID-19 |
| 21 | Le Roux G | 2020 | COVID-19: home poisoning throughout the containment period | Excluded | Was not performed in the context of COVID-19 |
| 22 | Makowska M | 2020 | Self-medication-related behaviors and Poland’s Covid-19 lockdown | Excluded | Was not performed in the context of COVID-19 |
| 23 | Onchonga D | 2020 | Assessing the prevalence of self-medication among healthcare workers before and during the 2019 SARS-CoV-2 (COVID-19) pandemic in Kenya | Excluded | Was not performed in the context of COVID-19 |
| 24 | Vidot DC | 2020 | The COVID-19 cannabis health study: Results from an epidemiologic assessment of adults who use cannabis for medicinal reasons in the United States | Excluded | Was not performed in the context of COVID-19 |
| 25 | Mauro A | 2020 | Infection control strategy and primary care assistance in Campania region during the national lockdown due to COVID-19 outbreak: the experience of two tertiary emergency centers | Excluded | Was not performed in the context of COVID-19 |
| 26 | Goodwin | 2020 | Psychological and behavioural responses to COVID-19: a China–Britain comparison | Excluded | Was not performed in the context of COVID-19 |
| 27 | Ahmed I | 202**0** | Behavioral preventive measures and the use of medicines and herbal products among the public in response to Covid-19 in Bangladesh:  A cross-sectional study | Excluded | Did not assess self-medication |
| 28 | Aqeel U | 2020 | Knowledge, attitudes, and practices toward coronavirus disease-19 infection among residents of delhi ncr, india: A cross-sectional survey based study | Excluded | Did not assess self-medication |
| 29 | Arasteh P | 2020 | A Surge in Methanol Poisoning Amid COVID-19 Pandemic: Why Is This Occurring? | Excluded | Did not assess self-medication |
| 30 | Brown JD | 2020 | Survey-reported medication changes among older adults during the SARS-CoV-2 (COVID-19) pandemic | Excluded | Did not assess self-medication |
| 31 | Charan J | 2020 | Use of Complementary and Alternative Medicine (CAM) and Home Remedies by COVID-19 Patients: A Telephonic Survey | Excluded | Did not assess self-medication |
| 32 | Chauhan V | 2020 | Proctoring Hydroxychloroquine Consumption for Health‑care  Workers in India Awaiting Revised National Guidelines | Excluded | Did not assess self-medication |
| 33 | Elbeddini A | 2020 | Role of Canadian pharmacists in managing drug shortage concerns amid the COVID-19 pandemic | Excluded | Did not assess self-medication |
| 34 | Haque M | 2020 | Availability and price changes of potential medicines and equipment for the prevention and treatment of covid-19 among pharmacy and drug stores in bangladesh; findings and implications | Excluded | Did not assess self-medication |
| 35 | Kamarli Altun H | 2021 | Evaluation of dietary supplement, functional food and herbal medicine use by dietitians during the COVID-19 pandemic | Excluded | Did not assess self-medication |
| 36 | Kristina SA | 2020 | The perception of role and responsibilities during covid-19 pandemic: A survey from indonesian pharmacists | Excluded | Did not assess self-medication |
| 37 | Nicholas T | 2020 | COVID-19 knowledge, attitudes and practices in a conflict affected area of the South West Region of Cameroon | Excluded | Did not assess self-medication |
| 38 | Vaduganathan M | 2020 | Prescription Fill Patterns for Commonly Used Drugs during the COVID-19 Pandemic in the United States | Excluded | Did not assess self-medication |
