# Supplementary material for: Self-medication practices to prevent or manage COVID-19: A systematic review
Source: PLoS One. 2021 Nov 2;16(11):e0259317. doi: 10.1371/journal.pone.0259317 (PMC8562851; doi:10.1371/journal.pone.0259317)
Supplement: Attachment — Submitted filename: PONE-D-21-21439.pdf [file pone.0259317.s004.pdf]

PLOS ONE

Self-medication practices for COVID-19: a systematic review

--Manuscript Draft--

**Manuscript Number:**	PONE-D-21-21439
**Article Type:**	Research Article
**Full Title:**	Self-medication practices for COVID-19: a systematic review
**Short Title:**	Self-medication practices for COVID-19
**Corresponding Author:**	Alvaro Taype-Rondan<br>Universidad San Ignacio de Loyola<br>Lima, PERU
**Keywords:**	COVID-19; SARS-CoV-2; Self Medication; Global Health; Pharmaceutical Preparations; Systematic Review
**Abstract:**	Objectives<br><br>As the COVID-19 pandemic progresses, many people may embrace self-medication, whose frequency and characteristics are important subjects of study in order to understand its dangers and design interventions. We conducted a systematic review to assess the self-medication frequency for the prophylaxis or management of COVID-19.<br><br>Methods<br><br>For this systematic review, we used different keywords and searched studies published in PubMed, Scopus, Web of Science, Embase, two preprints repositories, Google, and Google Scholar. We included studies which reported original data and assessed self-medication for prophylaxis of COVID-19 or management as main or secondary outcome. The risk of bias assessment was evaluated with the Newcastle-Ottawa Scale modified for cross-sectional studies.<br><br>Results<br><br>We found 11 studies that assessed self-medication for prevention or management of the COVID-19. Most of the studies did not detailed the questions used to assess self-medication nor the recall period. In the 7 studies that assessed self-medication for any reason, the self-medication frequencies ranged between 14.8% and 88.3%, and the most mentioned drugs were antibiotics, acetaminophen, chloroquine or hydroxychloroquine, vitamins, or supplements, ivermectin, ibuprofen, and antiasthmatics. Most of the studies had a risk of bias in the representativeness of the sample and the outcome assessment.<br><br>Conclusions<br><br>Self-medication frequency was heterogeneous, and the methods used in the included studies were unclear. Thus, more well-designed, and adequately reported studies in this regard are warranted.
**Order of Authors:**	Alvaro Quincho-Lopez
	Christeam A Benites-Ibarra
	Maryori M. Hilario-Gomez
	Renatta Quijano-Escate
	Alvaro Taype-Rondan
**Additional Information:**	
**Question**	**Response**
**Financial Disclosure**	The author(s) received no specific funding for this work.

Powered by Editorial Manager® and ProduXion Manager® from Aries Systems Corporation

Enter a financial disclosure statement that describes the sources of funding for the work included in this submission. Review the submission guidelines for detailed requirements. View published research articles from *PLOS ONE* for specific examples.<br><br>This statement is required for submission and **will appear in the published article** if the submission is accepted. Please make sure it is accurate.<br><br>**Unfunded studies**<br>Enter: *The author(s) received no specific funding for this work.*<br><br>**Funded studies**<br>Enter a statement with the following details:<br>• Initials of the authors who received each award<br>• Grant numbers awarded to each author<br>• The full name of each funder<br>• URL of each funder website<br>• Did the sponsors or funders play any role in the study design, data collection and analysis, decision to publish, or preparation of the manuscript?<br>• **NO** - Include this sentence at the end of your statement: *The funders had no role in study design, data collection and analysis, decision to publish, or preparation of the manuscript.*<br>• **YES** - Specify the role(s) played.<br><br>* typeset	
**Competing Interests**<br><br>Use the instructions below to enter a competing interest statement for this submission. On behalf of all authors, disclose any competing interests that could be perceived to bias this work—acknowledging all financial support and any other relevant financial or non-financial competing interests.<br><br>This statement is required for submission and **will appear in the published article** if the submission is accepted. Please make	The authors have declared that no competing interests exist.

Powered by Editorial Manager® and ProduXion Manager® from Aries Systems Corporation

sure it is accurate and that any funding sources listed in your Funding Information later in the submission form are also declared in your Financial Disclosure statement.<br><br>View published research articles from *PLOS ONE* for specific examples.<br><br>**NO authors have competing interests**<br><br>Enter: *The authors have declared that no competing interests exist.*<br><br>**Authors with competing interests**<br><br>Enter competing interest details beginning with this statement:<br><br>*I have read the journal's policy and the authors of this manuscript have the following competing interests: [insert competing interests here]*<br><br>* typeset	
**Ethics Statement**<br><br>Enter an ethics statement for this submission. This statement is required if the study involved:<br><br>• Human participants<br>• Human specimens or tissue<br>• Vertebrate animals or cephalopods<br>• Vertebrate embryos or tissues<br>• Field research<br><br>Write "N/A" if the submission does not require an ethics statement.<br><br>General guidance is provided below. Consult the submission guidelines for detailed instructions. **Make sure that all information entered here is included in the Methods section of the manuscript.**	N/A

**Format for specific study types**<br><br>**Human Subject Research (involving human participants and/or tissue)**<br>• Give the name of the institutional review board or ethics committee that approved the study<br>• Include the approval number and/or a statement indicating approval of this research<br>• Indicate the form of consent obtained (written/oral) or the reason that consent was not obtained (e.g. the data were analyzed anonymously)<br><br>**Animal Research (involving vertebrate animals, embryos or tissues)**<br>• Provide the name of the Institutional Animal Care and Use Committee (IACUC) or other relevant ethics board that reviewed the study protocol, and indicate whether they approved this research or granted a formal waiver of ethical approval<br>• Include an approval number if one was obtained<br>• If the study involved *non-human primates*, add *additional details* about animal welfare and steps taken to ameliorate suffering<br>• If anesthesia, euthanasia, or any kind of animal sacrifice is part of the study, include briefly which substances and/or methods were applied<br><br>**Field Research**<br><br>Include the following details if this study involves the collection of plant, animal, or other materials from a natural setting:<br>• Field permit number<br>• Name of the institution or relevant body that granted permission	
**Data Availability**<br><br>Authors are required to make all data underlying the findings described fully available, without restriction, and from the time of publication. PLOS allows rare exceptions to address legal and ethical concerns. See the PLOS Data Policy and FAQ for detailed information.	Yes - all data are fully available without restriction

Powered by Editorial Manager® and ProduXion Manager® from Aries Systems Corporation

A Data Availability Statement describing where the data can be found is required at submission. Your answers to this question constitute the Data Availability Statement and **will be published in the article**, if accepted.<br><br>**Important:** Stating 'data available on request from the author' is not sufficient. If your data are only available upon request, select 'No' for the first question and explain your exceptional situation in the text box.<br><br>Do the authors confirm that all data underlying the findings described in their manuscript are fully available without restriction?	
**Describe where the data may be found in full sentences. If you are copying our sample text, replace any instances of XXX with the appropriate details.**<br><br>• If the data are **held or will be held in a public repository**, include URLs, accession numbers or DOIs. If this information will only be available after acceptance, indicate this by ticking the box below. For example: *All XXX files are available from the XXX database (accession number(s) XXX, XXX.*).<br>• If the data are all contained **within the manuscript and/or Supporting Information files**, enter the following: *All relevant data are within the manuscript and its Supporting Information files.*<br>• If neither of these applies but you are able to provide **details of access elsewhere**, with or without limitations, please do so. For example:<br><br>*Data cannot be shared publicly because of [XXX]. Data are available from the XXX Institutional Data Access / Ethics Committee (contact via XXX) for researchers who meet the criteria for access to confidential data.*<br><br>*The data underlying the results presented in the study are available from (include the name of the third party*	All relevant data are within the paper and its Supporting Information files.

and contact information or URL).<br>• This text is appropriate if the data are owned by a third party and authors do not have permission to share the data.<br><br>* typeset	
Additional data availability information:	Tick here if your circumstances are not covered by the questions above and you need the journal’s help to make your data available.

Powered by Editorial Manager® and ProduXion Manager® from Aries Systems Corporation

Manuscript Click here to access/download;Manuscript;Manuscript.docx

Self-medication practices for COVID-19: a systematic review

Short title: Self-medication practices for COVID-19

Authors:

Alvaro Quincho-Lopez[1]

Christeam A. Benites-Ibarra[2]

Maryori M. Hilario-Gomez[1]

Renatta Quijano-Escate[3]

Alvaro Taype-Rondan[4]

Affiliations:

[1]Universidad Nacional Mayor de San Marcos, Sociedad Científica de San Fernando, Lima, Peru

[2]Sociedad Científica de Estudiantes de Medicina de la Universidad Nacional del Santa, Nuevo Chimbote, Ancash, Peru

[3]Universidad Nacional San Luis Gonzaga, Sociedad Científica de Estudiantes de Medicina de Ica, Ica, Peru

[4]Universidad San Ignacio de Loyola, Unidad de Investigación para la Generación y Síntesis de Evidencias en Salud, Lima, Peru

Funding:

This study was self-funded by the authors.

Corresponding author:

Alvaro Taype-Rondan

Universidad San Ignacio de Loyola, Unidad de Investigación para la Generación y Síntesis de Evidencias en Salud

Av. La Fontana 750, Lima, Peru

alvaro.taype.r@gmail.com

Abstract

Objectives: With the progression of the coronavirus disease 2019 (COVID-19) pandemic, several individuals may adopt self-medication. To understand the associated dangers and design interventions, it is important to study the ~~frequency~~ prevalence and characteristics of self-medication. We conducted a systematic review to assess the ~~frequency~~ of self-medication for the prophylaxis or management of COVID-19.

Methods: For this systematic review, we used different keywords and searched studies published in PubMed, Scopus, Web of Science, Embase, two preprint repositories, Google, and Google Scholar. We included studies that reported original data and assessed self-medication for the prophylaxis or management of COVID-19 as a main or secondary outcome. Risk of bias was assessed using the Newcastle–Ottawa Scale modified for cross-sectional studies.

Results: We identified 11 studies that assessed self-medication for the prevention or management of COVID-19. Most studies ~~neither~~ detailed the questions used to assess self-medication nor the recall period. In the seven studies that assessed self-medication for any reason, frequency of self-medication ranged between 14.8% and 88.3%, and the most mentioned drugs were antibiotics; acetaminophen; chloroquine or hydroxychloroquine; vitamins or supplements; ivermectin, ibuprofen, and antiasthmatics. Most studies had a risk of bias in the representativeness of the sample and assessment of outcome.

Conclusions: The frequency of self-medication was heterogeneous, and the methods used in the included studies were unclear. Thus, more well-designed and adequately reported studies in this regard are warranted.

Keywords: COVID-19, SARS-CoV-2, Self-Medication, Global Health, Pharmaceutical Preparations, Systematic Review

1 Introduction

According to the World Health Organization (WHO), self-medication is the administration of medications without consulting a health professional and without medical supervision [1]. Its prevalence varies with country [2–4], age group [5,6], and occupation [7].

With the progression of the coronavirus disease 2019 (COVID-19) pandemic, several drugs have been proposed as potential candidates for this disease [8], most of which are now known to have little or no benefit [9,10]. However, the fear of contracting the virus, low access to health services, and massive misinformation has led some people to employ self-medication, with expected direct and indirect consequences [11,12].

Previous studies have assessed the frequency and characteristics of self-medication for COVID-19 and have helped understand which useless or potentially hazardous drugs are being employed and which characteristics predispose individuals to a greater frequency of self-medication. Thus, in this systematic review, we aimed to assess the frequency of self-medication for the prophylaxis or management of COVID-19.

2 ~~Materials and~~ methods

This systematic review was reported according to the Preferred Reporting Items for Systematic Reviews and Meta-Analyses (Supplement Table S3) [13]. The study protocol has been registered at PROSPERO (CRD42021236191).

2.1 Data sources

On February 4th, 2021, we searched PubMed, Scopus, Web of Science, Embase, two preprint repositories (MedRxiv and SciELO Preprints), Google, and Google Scholar. No language restriction was applied. The search was restricted to include documents published in 2020 or 2021 (see S1 Table).

2.2 Inclusion criteria

We included original studies, either published in scientific journals or with full text available in preprint repositories, which reported original data and assessed the use of self-medication for the prophylaxis and/or management of COVID-19 as a main or secondary outcome. Self-medication was defined as the use of ~~drugs~~ w~~ithout a prescription from a health professional~~.

2.3 Study selection

Two authors (AQL and MHG) independently selected the studies. Screening included a review of the search results by title and abstract according to the inclusion criteria using the web program Rayyan (http://rayyan.qcri.org), followed by reviewing the full text of the relevant studies to determine whether they were appropriate for study inclusion. Discrepancies were consulted with another author (ATR) and resolved with consensus.

2.4 Data extraction

Two authors (CBI and RQE) independently extracted the relevant data for the review using a prepiloted Microsoft Excel spreadsheet. Again, any discrepancies were discussed with another author (ATR) and resolved with consensus. The following variables were assessed for each study: author, year of publication, country, study design, setting, population, sample technique, date of data collection, number of participants, age, sex, characteristics that may be related to self-medication, method of assessment of self-medication, results of frequency of self-medication, reasons for practicing self-medication (including who recommended such medication), from where the medication was obtained, and adverse events related to its use.

2.5 Risk-of-bias assessment

Two authors (CBI and RQE) independently evaluated the risk of bias for each included study using the Newcastle–Ottawa Scale (NOS) adapted for descriptive cross-sectional studies [14]. This scale comprises two criteria: the selection criteria (representativeness of the sample, sample size, and nonresponders) and the outcome criteria (outcome assessment and statistical tests). Any discrepancy was consulted with another author (ATR).

2.6 Statistical analysis

Because studies were performed in different populations and using different definitions of self-medication, we did not perform meta-analyses and decided to present the results of each study separately.

3 Results

3.1 Study selection

A total of 765 records were found in the database search. After the duplicates were eliminated, ~~we screened 227 records, of which we reviewed 46 full-text documents. We excluded 35 articles (S2~~ Table) and included 11 articles, all of which were cross-sectional studies (Fig. 1).

[Insert Figure 1]

Fig 1. Flow diagram of study selection.

3.2 Methodological quality of the included studies

In the risk-of-bias assessment using the NOS, ~~most studies~~ received 4 out of 5 stars; all studies except one [15] failed in the representativeness of the sample item of the selection criteria (Table 1). We considered the article by Sadio et al twice because of the different sampling methods used in that study (probability and nonprobability).

Table 1. Quality assessment of included studies using the Newcastle–Ottawa Scale (NOS) adapted for cross-sectional studies.

Study	Final score[6]	Representativeness of the sample[1]	Sample size[2]	Not respondents[3]	Outcome evaluation[4]	Statistical test[5]
Quispe-Cañari (2020)	4/5		★	★	★	★
Nasir (2020)	1/5				★	
Wegbom (2021)	3/5		★	★		★
Dare (2021)	2/5			★		★
Ahmed (2020)	0/5					
Mansuri (2020)	4/5		★	★	★	★

Zavala-Flores (2020)	1/5				★	
Sadio (2021)[7]	5/5	★	★	★	★	★
Sadio (2021)[8]	4/5		★	★	★	★
Miñan-Tapia (2020)	4/5		★	★	★	★
Kamarli Altun (2020)	~~2~~/5		★	★		★
Chauhan (2020)	0/5					

[1]Representativeness of the sample: One star was awarded to studies with random sampling.
[2]Sample size: One star was awarded to studies with a justified and satisfactory sample size.
[3]Non-respondents: If comparability between respondents' and nonrespondents' characteristics was established and the response rate was satisfactory, one star was assigned.
[4]Outcome evaluation: If the study explicitly defined how self-medication was defined and how long the recall period was, one star was assigned.
[5]Statistical test: One star was assigned if it was not a complex sample and the sample had been calculated correctly or if it was a complex sampling and the sampling was considered to determine the prevalence.
[6]The sum of stars of 1+2+3+4+5
[7]Probability sampling.
[8]Nonprobability sampling.

3.3 Study characteristics

The 11 included studies reported data on self-medication practices in eight different countries: three from Peru [16–18], two from Bangladesh [19,20], one from India [21], one from Togo [15], one from Nigeria [22], one from Uganda [23], one from Turkey [24], and one from the Kingdom of Saudi Arabia [25]. All studies were cross-sectional. Most of the studies (N = 7) were published as original reports, two were letters to the editor [17,21], and two were preprints reporting original data [18,23]. Studies were performed from March to August 2020.

Six studies were conducted targeting the general population, whereas one study was conducted in hospitalized patients with COVID-19 [17], one in health science students [18], one in health workers [21], one in dietitians [24], and one in workers from five sectors (health care, air transport, police, road transport, and informal) [15].

The 11 studies comprised 125–3,792 participants. All studies were performed in adults. Eight studies mentioned the mean or median age of the patients, which ranged from ~~21 to 60.3~~ years. The percentage of females ranged from 28.4% to 88.2% (Tables 2 and 3).

Table 2. Characteristics and findings of studies that assessed self-medication of any drug for any reason.

~~Study~~	Country (city)/ ~~date~~	Subjects (age, sex)	Survey application and self-medication definition	Frequency of self-medication for COVID-19 prevention or management (overall and most-used drugs)
**Studies performed in the general population:**				
~~Quispe-Cañari (2021)~~	Perú/June 2020	Adults (median age: 23 years, female: 54.5%), N = 3792	• Open online survey (convenience sampling)<br>• Respondents were asked to indicate whether they consumed any of the following drugs: acetaminophen, ibuprofen, azithromycin, hydroxychloroquine, penicillin, or antiretrovirals (for any reason)<br>• Recall period: during the COVID-19 lockdown	• **Overall:** 33.4% for any reason<br>• **For any reason:** acetaminophen (27.0%), ibuprofen (7.4%), azithromycin (4.8%), penicillin (2.3%), antivirals (1.6%), hydroxychloroquine (0.7%)
Nasir (2020)	Bangladesh (Dhaka city)/from April to June 2020	Adults (age: 50.5% between 45 and 54 years, female: 55.3%), N = 626	• Open online survey (convenience sampling)<br>• Self-medication was defined as taking drugs without prescription<br>• Recall period: during the COVID-19 outbreak	• **Overall:** 88.3% for any reason<br>• **For COVID-19 management:** ivermectin (77.2%), azithromycin (54.2%), montelukast (43.1%), calcium supplements (41.4%), doxycycline (40.3%), hydroxychloroquine (20.4%)
Wegbom (2021)	Nigeria/from June to July 2020	Adults (mean age: 42.2 years, female: 57.1%), N = 461	• Open online survey (convenience sampling)<br>• Self-medication was defined as taking drugs intended to prevent/treat COVID-19 without prescription by medically qualified personnel (for either prevention or management)<br>• Recall period: not detailed	• **Overall:** 41% for prevention or management<br>• **For COVID-19 prevention or management:** Vitamin C or multivitamin (21.3%), antimalarial drugs other than hydroxychloroquine and chloroquine (19.3%), amoxicillin (10.2%), ciprofloxacin (6.1%), herbal products (4.1%), metronidazole (3.5%), erythromycin (2.2%), hydroxychloroquine or chloroquine (1.3%)
Ahmed (2020)	Bangladesh/from June to July 2020	Adults (mean age: 30.8 years, female: 38.6%), N = 1222	• In-person and online survey (purposive sampling to include participants of all ages and diverse educational and socioeconomic backgrounds)<br>• Self-medication defined as the use of medicines and herbal products/foods as preventive and curative measures against COVID-19<br>• Recall period: not detailed	• **Overall:** 14.8% for prevention or management<br>• **As a preventive measure:** arsenicum album (4.5%), vitamin supplements (4.0%), mineral supplements (2.9%), acetaminophen (2.4%), antihistamines (1.7%), antiasthmatics (1.3%), ivermectin (0.8%)<br>• **To manage symptoms:** acetaminophen: (13.6%), antihistamines (6.0%), antibiotics (3.9%), mineral supplements (3.9%), antiasthmatics (3.6%), vitamin supplements (3.0%), ivermectin (2.2%)
Dare (2020) - preprint	Uganda (Western region)/from June to August 2020	Adults from Uganda western cities (age: 43.8% between 25 and 34 years, female: 45.2%), N = 272	• In-person and online survey (convenience sampling)<br>• Self-medication assessment was not detailed<br>• Recall period: during COVID-19 pandemic	• **Overall:** 57% for any reason
**Studies performed in specific populations**				
Sadio (2021)	Togo (Lomé)/from April to May 2020	Adults from any of these five sectors: health care, air transport, police, road transport, and informal (median age: 36 years, female: 28.4%), N = 955	• In-person survey through recruitment, open invitation, and random sampling (two or three stages)<br>• Self-medication was defined as the use of medicines/drugs alleged to treat or prevent COVID-19 without a physician order<br>• Recall period: 2 weeks	• **Overall:** 34.2% for prevention or management<br>• Vitamin C (27.6%), traditional medicine (10.2%), chloroquine/hydroxychloroquine (2.0%), azithromycin (1.2%)
Miñan-Tapia (2020) - preprint	Peru (Tacna)/from June to July 2020	Undergraduate students of health-related careers from two universities (median age: 21 years, female: 69.1%), N = 718	• Online, convenience sampling<br>• Self-medication was defined as using any of the prespecified 14 medicines for COVID-19 without medical prescription<br>• Recall period: during the past 3 months	• **Overall:** 51.3% for any reason<br>• Acetaminophen (41.4%), ibuprofen (21.1%), dexamethasone (10.7%), aspirin (8.6%), azithromycin (4.9%)

3.4 Definition of self-medication

All studies assessed self-medication through self-report. However, the questions used to assess self-medication were not explicitly mentioned in 9 of the 11 studies. Moreover, four studies did not clearly detail the recall period for self-medication: it was considered as anytime during the COVID-19 pandemic in four studies, in the past 2 weeks in one study, in the past 3 months in one study, and since the COVID-19 diagnosis in another study that assessed hospitalized patients (Tables 2 and 3).

3.5 Frequency of self-medication and most frequently used drugs

Seven studies assessed self-medication for any reason: five in the general population (with a range of frequency of self-medication between 14.8% and 88.3%) and two in specific populations (with a frequency of self-medication of 34.2% in adults who worked in any of the five assessed sectors and 51.3% in undergraduate students of health-related careers). Of these seven studies, only two specified which question was asked [16,18], whereas the other five studies did not detail whether participants could report self-medication only for a prespecified list of drugs or whether it was an open question.

Of the seven studies that assessed self-medication for any reason, six detailed the list of drugs reported by the participants. The reported drugs were different across the six studies: six studies reported the consumption of antibiotics; three of acetaminophen; four of chloroquine or hydroxychloroquine; four of vitamins or supplements; two of ivermectin; two of ibuprofen; two of antiasthmatics; and one of other drugs. In addition, consumption preferences varied across studies. For example, when drugs were sorted in the order of highest to lowest consumption, vitamins/supplements occupied the first place in two studies, fourth place in one study, and in the last study, second place as a preventive measure and sixth place to manage symptoms (Table 2).

The other four studies assessed self-medication of specific drugs or for specific reasons: one of these studies was performed in the general population of Saudi Arabia and found that 35.1% of the surveyed individuals used self-medication for fever [25]. Another study found that 33.9% of patients with COVID-19 in a Peruvian hospital used self-medication since their diagnosis (mainly with antibiotics, ivermectin, and corticosteroids) [17]. The other two studies assessed the use of a specific

drug for COVID-19 prevention: one study found that 94.5% of the surveyed dietitians in Turkey consumed dietary supplements for COVID-19 prevention [24], whereas the other study found that 46.4% of the surveyed doctors and nurses in India used hydroxychloroquine for the prevention of COVID-19 [21] (Table 3).

Table 3. Characteristics and findings of studies that assessed self-medication of specific drugs or for specific reasons.

Study	Country (city)/ date	Subjects (age, sex)	Survey application and self-medication definition	Frequency of self-medication for COVID-19 prevention or management (overall and most-used drugs)
**Studies that assessed the use of drugs for specific reasons:**				
Mansuri (2020)	Kingdom of Saudi Arabia/from March to April 2020	Adults (age: 60.8% <40 years, female: 60.3%), N = 388	• Online electronic survey, convenience sampling<br>• Self-medication was assessed using the question, "Are you self-medicating for fever?"<br>• Recall period: during COVID-19 pandemic	• **Overall:** 35.1% to manage fever
Zavala-Flores (2020)– letter to the editor	Peru (Lima)/June 2020	Hospitalized adults with COVID-19 (Mean age: 60.3, Female: 30.3%), N = 132	• Authors included all patients hospitalized during a certain week<br>• Self-medication was defined as using some type of COVID-19 medication before hospitalization without medical prescription<br>• Recall period: since the COVID-19 diagnosis	• **Overall:** 33.9% for management<br>• Antibiotics (28.3%), ivermectin (20.7%), corticosteroids (17.0%), acetaminophen (12.3%), aspirin (4.7%), NSAID (4.7%), hydroxychloroquine (0.9%), others (1.9%)
**Studies that assessed the use of specific drugs:**				
Kamarli Altun (2020)	Turkey (Antalya)/from May to June 2020	Dietitians graduated from Turkish universities (mean age: 30.6 years, female: 88.2%), N = 550	• Online, sample with a known universe<br>• Self-medication was defined as taking dietary supplements for COVID-19 prevention<br>• Recall period: not detailed	**Overall:** 94.5% took dietary supplements for COVID-19 prevention
Chauhan (2020)– letter to the editor	India/dates not specified	Doctors and nurses (mean age: 37.7 years, female: 29.6%), N = 125	• Online, voluntarily completed<br>• Self-medication was defined as taking hydroxychloroquine for COVID-19 prevention<br>• Recall period: not detailed	**Overall:** 46.4% used hydroxychloroquine for COVID-19 prevention

NSAID, nonsteroidal anti-inflammatory drug

3.6 Reasons to practice self-medication

Five of the 11 studies mentioned the reasons for self-medication. However, this was assessed heterogeneously across studies. Thus, one study referred to the symptoms, another suggested that participants used self-medication based on the recommendations of national groups, and the other three studies mentioned a plethora of reasons, including the fear of stigmatization, fear of quarantine,

affordability, convenience of self-medication, patients believing that the symptoms were not serious, and suggestions of family and friends (Table 4).

3.7 Who suggested the practice of self-medication and from where were the medications obtained?

Three studies assessed who suggested the use of self-medications. One study reported that patients received advice from family, friends, or relatives (40.1%) or relied on their own knowledge (32.4%). Media (print, digital, and social) was also a major source of information. However, only 25% of the participants sought advice from doctors, whereas 18% consulted with pharmacists [19]. Another study reported that patients were advised by a doctor, pharmacist, literature, medicine company, friends, or TV/radio programs [24]. The third study indicated that patients relied on the Indian Council of Medical Research guideline (71.4%) or advice from a senior fellow or colleague (23.8%) [21].

Only one study [22] described from where the patients obtained their self-medications. The most common sources for obtaining medications were the pharmacy (73.9%), patent medicine vendor (23.6%), hospital (7.6%), hawkers (4.5%), and faith-based outlets and herbalists (2.1%, each).

Table 4. Reasons to practice self-medication, who suggested its practice, and from where such medications were obtained.

Study	Reasons to practice self-medication	Suggested by	Obtained from
Nasir (2020)	Fever (37.6%), throat pain (28.8%), dry cough (14.2%), loss of smell (9.2%), loss of taste (3.5%), body ache (5.0%), and rarely diarrhea (1.7%)	NR	NR
Chauhan (2020)	The article suggested that self-medication was based on the recommendations made by the Indian Council of Medical Research on the empirical use of hydroxychloroquine	Indian Council of Medical Research guideline (71.4%), advice from senior fellow or colleague (23.8%)	NR
Wegbom (2021)	Fear of stigmatization or discrimination (79.5%), fear of quarantine or self-isolation (77.3%), fear of infection or contact with a suspected or known COVID-19 patient (76.3%), delay in receiving treatment at health facilities" (55.6%), influence of friends" (55.2%), social media (54.3%), nonavailability of drugs for COVID-19 treatment in the health facilities (53%), emergency illness (49.1%), delay in getting hospital services (28.1%), distance to the health facility (23%), proximity of the pharmacy (21%), nonavailability of medicine in a health facility (19.3%), and health facility charges (15.3%)	NR	Pharmacy (73.9%), patent medicine vendor (23.6%), hospital (7.6%), hawkers (4.5%), faith-based outlets and herbalists (2.1%, each)

Dare (2020)	Self-medication is affordable (37%) and convenient (32%), lack the means to get to the health facility/hospital (15%), fear of being diagnosed COVID-19 positive (9%), fear of visiting health facility or hospital (7%)	NR	NR
Miñan-Tapia (2020)	Believing that the symptoms were not so serious to go to a doctor (64.3%), referring to family/friends that are nonmedical health professionals (34.9%), and owing to economic reasons or use of over-the-counter medications (34.9%)	NR	NR
Ahmed (2020)	NR	Advice of family, friends, or relatives (40.1%) and their own knowledge (32.4%); advice from doctors (25%); advice from pharmacists (18%)	NR
Kamarli Altun (2020)	NR	Advise of a doctor, pharmacist, literature, medicine company, friends, or TV/radio programs	NR

NR, not reported

3.8 Adverse events due to self-medication for COVID-19

Only three studies reported whether adverse events occurred. One study found that 11.7% patients presented with side effects related to self-medication in the past 3 months [18]. Nonetheless, this study did not specify the adverse effects that occurred. The second study reported body rash (23.1%) followed by worsened condition (17.3%), yellowish eyes (7.7%), swollen face (3.8%), and vomiting of blood and severe diarrhea (5.8%) [22]. The third study reported only gastrointestinal upset [21].

4 Discussion

Because self-medication is a global phenomenon that may involve health risks at both the individual and community level, it is important to assess its frequency [26,27]. Previous studies have found that self-medication is a common practice, with a prevalence of 53%, 44%, and 53.6% (recall period: a single day to 6 months) according to systematic reviews conducted in Iran [2], Ethiopia [3], and India [4], respectively.

Although studies were performed in eight different countries, the lack of studies in some regions such as North America, the Middle East, North Africa, and Oceania is evident. It is expected that the intercountry differences in drug promotion, regulations, and the possibility of accessing some drugs

without a prescription influence the self-medication patterns [28,29]. In this review, 10 of the 11 studies were performed in low- and middle-income countries. However, the results do not suggest that self-medication is higher in low- and middle-income countries than in other countries.

Some of the included studies assessed symptomatic drugs such as acetaminophen or nonsteroidal anti-inflammatory drugs, which are some of the symptomatic medications used for COVID-19, along with several other diseases. In addition, studies have found an important, although heterogeneous, frequency of self-medication use with antibiotics, chloroquine or hydroxychloroquine, vitamins or supplements, ivermectin, and antiasthmatics. As stated by the current guidelines, none of these drugs has proven to have any benefit in the prevention or the ambulatory management of COVID-19 [30,31].

In fact, the adverse effects of some of these drugs are of great concern, such as antibiotic resistance due to extensive antibiotic use, bleeding caused by aspirin use, inhibition of the immune system caused by corticosteroids, or arrhythmia caused by hydroxychloroquine. Some healthcare organizations have issued statements on self-medication. For instance, the WHO recognizes that "successful" (i.e., beneficial) self-medication can be achieved in many countries only by improving individuals' knowledge and education level in such a way that the potential damages of this practice can be avoided [1]. Similarly, the International Pharmaceutical Federation, in tandem with the World Self-Medication Industry [32] as well as the World Medical Association [33], emphasizes the responsible use of nonprescription drugs.

As found in previous systematic reviews [5,6], studies tended to use different questions to assess self-medication or they simply did not specify the question used. Moreover, studies have established different recall periods, and even those that assessed self-medication since the beginning of the pandemic would have different time frames depending on the month in which they were performed. This prevents direct comparisons between studies and the subsequent meta-analysis of their results [34], which adds a potential source of bias that affects the internal validity of the results [34,35].

Most studies seemed to have been performed using convenience sampling. This may lead to misleading results because the results cannot be extrapolated to the general population owing to the nonprobability sampling used as well as the risk of sampling and nonresponder biases [36]. Accordingly, in this review, the representativeness of the sample domain was the worst-rated item in the risk-of-bias assessment.

5 Conclusion

We identified 11 studies that assessed the use of self-medication for the prevention or management of COVID-19. Most studies neither detailed the questions used to assess self-medication nor the recall period. In the seven studies that assessed self-medication for any reason, the frequencies of self-medication ranged from 14.8% to 88.3%, and the most mentioned drugs were antibiotics; acetaminophen; chloroquine or hydroxychloroquine; vitamins or supplements; ivermectin; ibuprofen; and antiasthmatics. Most studies had a risk of bias in the representativeness of the sample and assessment of the outcomes. Thus, more well-designed and adequately reported studies are warranted in this regard.

Acknowledgments

None.

References

1. World Health Organization. Guidelines for the regulatory assessment of medicinal products for use in self-medication. 2000 [cited 2021 Apr 10]. Available: https://apps.who.int/iris/handle/10665/66154

2. Azami-Aghdash S, Mohseni M, Etemadi M, Royani S, Moosavi A, Nakhaee M. Prevalence and cause of self-medication in Iran: A systematic review and meta-analysis article. Iran J Public Health. 2015;44: 1580–1593.

3. Sisay M, Mengistu G, Edessa D. Epidemiology of self-medication in Ethiopia: A systematic review and meta-analysis of observational studies. BMC Pharmacol Toxicol. 2018;19: 56.

4. Rashid M, Chhabra M, Kashyap A, Undela K, Gudi SK. Prevalence and Predictors of Self-Medication Practices in India: A Systematic Literature Review and Meta-Analysis. Curr Clin Pharmacol. 2019;15: 90–101.

5. Shehnaz SI, Agarwal AK, Khan N. A systematic review of self-medication practices among adolescents. J Adolesc Heal. 2014;55: 467–483.

6. Jerez-Roig J, Medeiros LFB, Silva VAB, Bezerra CLPAM, Cavalcante LAR, Piuvezam G, et al. Prevalence of Self-Medication and Associated Factors in an Elderly Population: A Systematic Review. Drugs and Aging. 2014;31: 883–896.

7. Behzadifar M, Behzadifar M, Aryankhesal A, Ravaghi H, Baradaran HR, Sajadi HS, et al. Prevalence of self-medication in university students: Systematic review and meta-analysis. East Mediterr Heal J. 2020;26: 846–857.

8. Sanders JM, Monogue ML, Jodlowski TZ, Cutrell JB. Pharmacologic Treatments for Coronavirus Disease 2019 (COVID-19): A Review. JAMA - J Am Med Assoc. 2020;323: 1824–1836.

9. Lam S, Lombardi A, Ouanounou A. COVID-19: A review of the proposed pharmacological treatments. Eur J Pharmacol. 2020;886: 173451.

10. WHO Solidarity Trial Consortium. Repurposed Antiviral Drugs for Covid-19 — Interim WHO Solidarity Trial Results. N Engl J Med. 2021;384: 497–511.

11. Tasnim S, Hossain M, Mazumder H. Impact of rumors and misinformation on COVID-19 in Social Media. J Prev Med Public Heal. 2020;53: 171–174.

12. Onchonga D, Omwoyo J, Nyamamba D. Assessing the prevalence of self-medication among healthcare workers before and during the 2019 SARS-CoV-2 (COVID-19) pandemic in Kenya. Saudi Pharm J. 2020;28: 1149–1154.

13. Moher D, Shamseer L, Clarke M, Ghersi D, Liberati A, Petticrew M, et al. Preferred reporting items for systematic review and meta-analysis protocols (prisma-p) 2015 statement. Syst Rev. 2015;4: 1.

14. Herzog R, Álvarez-Pasquin MJ, Díaz C, Del Barrio JL, Estrada JM, Gil Á. Are healthcare workers intentions to vaccinate related to their knowledge, beliefs and attitudes? A systematic review. BMC Public Health. 2013;13: 154.

15. Sadio A, Gbeasor-Komlanvi F, KONU R, Bakoubayi A, Tchankoni M, Bitty-Anderson A, et al. Assessment of self-medication practices in the context of Covid-19 outbreak in Togo. BMC Public Health. 2021;21: 58.

16. Quispe-Cañari JF, Fidel-Rosales E, Manrique D, Mascaró-Zan J, Huamán-Castillón KM, Chamorro–Espinoza SE, et al. Self-medication practices during the COVID-19 pandemic among the adult population in Peru: A cross-sectional survey. Saudi Pharm J. 2021;29: 1–11.

17. Zavala-Flores E, Salcedo-Matienzo J. Medicación prehospitalaria en pacientes hospitalizados por COVID-19 en un hospital público de Lima-Perú. Acta Medica Peru. 2020;37: 393–395.

18. Miñan-Tapia A, Conde-Escobar A, Calderon-Arce D, Cáceres-Olazo D, Peña-Rios AJ, Carlos Donoso-Romero R. Factores asociados a la automedicación con fármacos relacionados a COVID-19 en estudiantes de ciencias de la salud de una ciudad peruana. SciELO Preprints [Preprint]. 2020 [cited 2021 May 15]. DOI: 10.1590/SciELOPreprints.1225

19. Ahmed I, Hasan M, Akter R, Sarkar BK, Rahman M, Sarker MS, et al. Behavioral preventive measures and the use of medicines and herbal products among the public in response to Covid-19 in Bangladesh: A cross-sectional study. PLoS One. 2020;15: e0243706.

20. Nasir M, Chowdhury ASMS, Zahan T. Self-medication during COVID-19 outbreak: a cross sectional online survey in Dhaka city. Int J Basic Clin Pharmacol. 2020;9: 1325–1330.

21. Chauhan V, Galwankar S, Raina S, Krishnan V. Proctoring hydroxychloroquine consumption for health-care workers in india as per the revised national guidelines. J Emergencies, Trauma Shock. 2020;13: 172–173.

22. Wegbom AI, Edet CK, Raimi O, Fagbamigbe AF, Kiri VA. Self-Medication Practices and Associated Factors in the Prevention and/or Treatment of COVID-19 Virus: A Population-Based Survey in Nigeria. Front Public Heal. 2021;9: 606801.

23. Dare S, Eze E, Isaac E, Usman I, Ssempijja F, Bukenya E, et al. COVID-19 pandemic and behavioural response to self-medication practice in Western Uganda. medRxiv: 2021.01.02.20248576v1 [Preprint]. 2020 [cited 2021 May 15]. Available from: https://www.medrxiv.org/content/10.1101/2021.01.02.20248576v1

24. Kamarli Altun H, Karacil Ermumcu MS, Seremet Kurklu N. Evaluation of Dietary Supplement, Functional Food and Herbal Medicine Use by Dietitians during the COVID-19 Pandemic. Public Health Nutr. 2020;24: 861–869.

25. Mansuri FMA, Zalat MM, Khan AA, Alsaedi EQ, Ibrahim HM. Estimating the public response to mitigation measures and self-perceived behaviours towards the COVID-19 pandemic. J Taibah Univ Med Sci. 2020;15: 278–283.

26. Bennadi D. Self-medication: A current challenge. J Basic Clin Pharm. 2014;5: 19.

27. Hughes CM, Mcelnay JC, Fleming GF. The benefits and risks of self-medication. Drug Saf.

2001;14: 1027–1037.

28. Leelavanich D, Adjimatera N, Broese Van Groenou L, Anantachoti P. Prescription and non-prescription drug classification systems across countries: Lessons learned for thailand. Risk Manag Healthc Policy. 2020;13: 2753–2768.

29. Morgan DJ, Okeke IN, Laxminarayan R, Perencevich EN, Weisenberg S. Non-prescription antimicrobial use worldwide: A systematic review. Lancet Infect Dis. 2011;11: 692–701.

30. World Health Organization. Therapeutics and COVID-19: living guideline. 2021 [cited 2021 May 15]. Available: https://www.who.int/publications/i/item/WHO-2019-nCoV-therapeutics-2021.1

31. Infectious Diseases Society of America (IDSA). IDSA Guidelines on the Treatment and Management of Patients with COVID-19. 2021 [cited 2021 May 15]. Available: https://www.idsociety.org/practice-guideline/covid-19-guideline-treatment-and-management/

32. Joint Statement by the International Pharmaceutical Federation and The World Self-Medication Industry. Responsible Self-Medication. 1999 [cited 2021 Apr 20]. Available: https://www.fip.org/file/1484

33. World Medical Association. WMA Statement on self-medication. 2017 [cited 2021 Apr 10]. Available: https://www.wma.net/policies-post/wma-statement-on-self-medication/

34. Limaye D, Limaye V, Krause G, Fortwengel G. A systematic review of the literature on survey questionnaires to assess self-medication practices. Int J Community Med Public Heal. 2017;4: 2620–2631.

35. Stull DE, Leidy NK, Parasuraman B, Chassany O. Optimal recall periods for patient-reported outcomes: Challenges and potential solutions. Curr Med Res Opin. 2009;25: 929–942.

36. Andrade C. The Limitations of Online Surveys. Indian J Psychol Med. 2020;42: 575–576.

Supporting information captions

S1 Checklist. PRISMA 2009 checklist.

S1 Table. Search strategies.

S2 Table. List of articles excluded after full-text review.

Figure 1

Click here to access/download;Figure;Fig 1.JPG

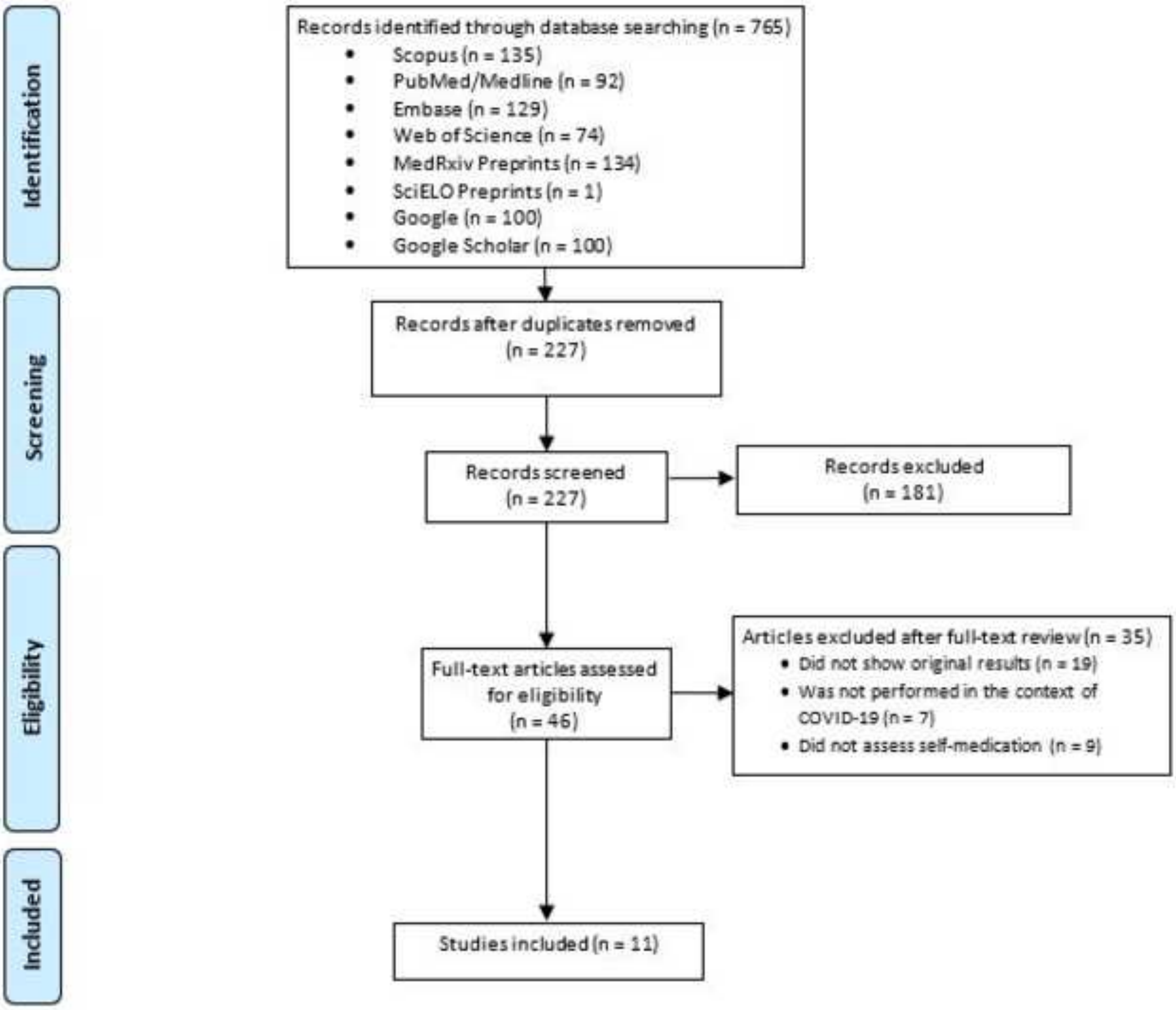

Click here to access/download
Supporting Information
S1 Checklist. PRISMA 2009 checklist..doc

S1 Table

Click here to access/download
Supporting Information
S1 Table. Search strategies..docx

S2 Table

Click here to access/download
Supporting Information
S2 Table. List of articles excluded after full-text review..docx
