# Supplementary material for: Self-medication practices to prevent or manage COVID-19: A systematic review
Source: PLoS One. 2021 Nov 2;16(11):e0259317. doi: 10.1371/journal.pone.0259317 (PMC8562851; doi:10.1371/journal.pone.0259317)
Supplement: Attachment — Submitted filename: PONE-D-21-21439_R1.pdf [file pone.0259317.s006.pdf]

PLOS ONE

Self-medication practices to prevent or manage COVID-19: a systematic review

--Manuscript Draft--

**Manuscript Number:**	PONE-D-21-21439R1
**Article Type:**	Research Article
**Full Title:**	Self-medication practices to prevent or manage COVID-19: a systematic review
**Short Title:**	Self-medication practices for COVID-19
**Corresponding Author:**	Alvaro Taype-Rondan<br>Universidad San Ignacio de Loyola<br>Lima, PERU
**Keywords:**	COVID-19; SARS-COV-2; Self Medication; global health; Pharmaceutical Preparations; Systematic Review
**Abstract:**	Background: Previous studies have assessed the prevalence and characteristics of self-medication in COVID-19. However, no systematic review has summarized their findings.<br>Objective: We conducted a systematic review to assess the prevalence of self-medication to prevent or manage COVID-19.<br>Methods: We used different keywords and searched studies published in PubMed, Scopus, Web of Science, Embase, two preprint repositories, Google, and Google Scholar. We included studies that reported original data and assessed self-medication to prevent or manage COVID-19. The risk of bias was assessed using the Newcastle–Ottawa Scale (NOS) modified for cross-sectional studies.<br>Results: We identified eight studies, all studies were cross-sectional, and only one detailed the question used to assess self-medication. The recall period was heterogeneous across studies. Of the eight studies, seven assessed self-medication without focusing on a specific symptom: four performed in the general population (self-medication prevalence ranged between <4% - 88.3%) and three in specific populations (range: 33.9% to 51.3%). In these seven studies, the most used medications varied widely, including antibiotics, chloroquine or hydroxychloroquine, acetaminophen, vitamins or supplements, ivermectin, and ibuprofen. The last study only assessed self-medication for fever due to COVID-19. Most studies had a risk of bias in the “representativeness of the sample” and “assessment of outcome” items of the NOS.<br>Conclusions: Studies that assessed self-medication for COVID-19 found heterogeneous results regarding self-medication prevalence and medications used. More well-designed and adequately reported studies are warranted to assess this topic.
**Order of Authors:**	Alvaro Quincho-Lopez
	Christeam A Benites-Ibarra
	Maryori M. Hilario-Gomez
	Renatta Quijano-Escate
	Alvaro Taype-Rondan
**Response to Reviewers:**	Dear editor and reviewers,<br><br>We have carefully considered the comments and have revised the application accordingly. We believe these comments will improve our manuscript. Our responses are presented below each of the reviewer's comments.<br><br>Reviewer #1<br><br>•R1C1: Needs an extensive language editing.<br>oWe agree with the recommendation, and a translator has checked the revised version of this manuscript.

Powered by Editorial Manager® and ProduXion Manager® from Aries Systems Corporation

	•R1C2: Introduction doesn't give enough back-ground information about the topic. It should have included a background on burden of COVID 19, self-medication, rationale/justification to conduct this review, availability of previously published review article.<br>oWe agree with the recommendation; we added the following text in the second and third paragraphs of the introduction:<br>Line 36-40: "This may lead to unintended consequences, such as adverse events, unnecessary expenses, delay in attending professional evaluation, masking of symptoms, and drug interactions [17–19]. Self-medication prevalence varies according to several factors, such as the methodology used to assess self-medication [20], the population characteristics [20–22], and across different countries and contexts [23–25]."<br>Line 41-43: "Previous studies have assessed the prevalence and characteristics of COVID-19 self-medication, reporting the use of several medications, herbal products, and dietary supplements as treatment or prevention for COVID-19 [26]. However, to date, no systematic review has summarized their findings."<br><br>•R1C3: Line 36-37, is the objective of this review just assessing the frequency of self-medication for COVID-19? Many things are mentioned in the result section. Please include all the specific objectives of this review at the end of the introduction section.<br>oWe agree with the recommendation and added these items to the objective (end of the introduction), as following:<br>Line 44-46: "we aimed to assess the prevalence of self-medication for the prevention or management of COVID-19. In addition, we assessed the type of medication used, reasons to practice self-medication, from where such medications were obtained, and adverse events due to its practice."<br><br>•R1C4: Better if 'frequency' is replaced by 'prevalence' throughout the document.<br>oWe agree with the recommendation and replaced "frequency" with "prevalence" throughout the manuscript.<br><br>•R1C5: Line 50-51, in the inclusion criteria you gave the definition of self- medication while you said the difference in definition is one of the limitations that hinder you from doing meta-analysis. so, remove the definition in the inclusion criteria. Ok to retain it in the introduction.<br>oIn order to clarify this, we have reformulated the text at the end of the "inclusion criteria" subheading of the methods, as follows:<br>Line 63-65: "We considered that a study assessed the use of self-medication in any of the following situations: 1) the study reported explicitly that self-medication was assessed (regardless of the definition used), or 2) study assessed the use of medications without medical prescription."<br><br>•R1C6: Include the following in the methods section, key words used for searching.<br>oThank you for your observation. The keywords used for the search in each database are available in the supplementary material (S1 Table). This is mentioned now in the second paragraph of the Methods:<br>Line 52-53: "Search strategies were constructed a priori using different terms related to 'COVID-19' and 'self-medication' (Supplementary Table S1)."<br><br>•R1C7: In your inclusion criteria nothing is said about the study participants in the retrieved studies (age (adult, children, or all), COVID status (positive, negative unknown)), region or geographic location where the studies were conducted, publication status (do you consider grey literature?).<br>oWe agree with the recommendation. We have added the following in the "inclusion criteria" subsection of the Methods:<br>Line 61-62: "in participants of any age and from any location."<br>oAlso, we have added the following in the "Data sources" subsection:<br>Line 54-55: "…and grey literature sources (Google and Google Scholar)."<br><br>•R1C8: 'Drug' and 'medication' are used interchangeably in the document, be consistent. I prefer 'medication' than 'drug' in your context.<br>oWe agree with the recommendation and replaced 'drug' by 'medication' throughout the manuscript where appropriate.

•R1C9: No language restriction was applied. Do you get non-English articles? If so how or who did the translation? It should be mentioned in the methods section.
oThanks for the observation. In order to clarify this, we have added the following to the "Data sources" subsection of the Methods:
Line 55-56: "We planned to hire a translation service if any study written in a language other than English or Spanish was found."

Comments from the PDF file:

•R1C10: please avoid 'we' and express in passive form.
oWe agree. We rephrased in passive from the first paragraph of the Results, as follows:
Line 93-95: "227 records were screened, of which 46 documents were assessed in full-text. Lastly, 38 papers were excluded (which are detailed in Supplementary Table S2), and 8 papers were included"

•R1C11: is this mean or median.
oThanks for the observation. To clarify this, we have improved the text of the third paragraph of the "Study characteristics" subsection of the results, as follows:
Line 139-141: "Five studies mentioned the summary measure (mean or median) of the participants' age. Such summary measure (either mean or median) ranged from 21 to 60.3 years."

•R1C12: should this study (Quispe-Cañari et al.) included in this review? b/c it doesn't assess self-medication for COVID-19.
oThanks for the observation. However, the study of Quispe Cañari et al. assessed self-medication for COVID-19 prevention and management (table 2 of the results of the study, link: https://www.ncbi.nlm.nih.gov/pmc/articles/PMC7832015/). These are the results that we took into account.

•R1C13: is this for specific reason? no difference with studies done by Wegbom, sadio, ahmed...
oWe agree. We moved Zavala-Flores et al. to the group of studies that assessed self-medication without restrictions. Also, we merged tables 2 and 3.

•R1C14: if you expect that self-medication in LMIC is higher you have to first back it up with an evidence (reference). then you can mention however...
oThanks for the suggestion. To clarify this, in the third paragraph of the discussion now it says:
"While self-medication may be higher in such countries, due to the conditions and structure of the health system [40], it is not possible to make solid comparisons due to the heterogeneity in the self-medication assessment across studies."

40.- Torres NF, Chibi B, Middleton LE, Solomon VP, Mashamba-Thompson TP. Evidence of factors influencing self-medication with antibiotics in low and middle-income countries: a systematic scoping review. Public Health. 2019;168: 92–101. doi:10.1016/j.puhe.2018.11.018

•R1C15: inappropriate word choice (symptomatic)
oWe agree. We added the following (line 210-212): "Some of the included studies assessed over-the-counter medications such as acetaminophen or nonsteroidal anti-inflammatory drugs, which are used for the symptom management of COVID-19, along with several other diseases".

Reviewer #2

Introduction

•R2C1: Paragraph 1. The introduction part could be written with more information. For example, the authors may explain more on the examples of prevalence difference between countries, age group and occupation, in terms of the highest self-medication practices. A more thorough definition on self-medication would also be very useful, as

this is the main focus of this paper, yet it is still unclear as it is only briefly mentioned in the introduction.
•R2C2: Paragraph 2. It would also be good if the authors described on the examples of drugs that has been proposed as the potential candidate for Covid-19, and the outcome of such treatments. The examples of direct and indirect consequences can also be further explained here, to make the introduction more informative.
•R2C3: Paragraph 3. Some description on the examples of medication used as self-treatment and indication in Covid would also be informative and will make the introduction more dynamic.
oWe agree with these 3 recommendations. Accordingly, we added the following text in the first paragraph of the introduction:
Line 25-32: "With the progression of the COVID-19 pandemic, several medications have been proposed as potential candidates for this disease [1], most of which resulted in little or no benefit for the patients [2,3] or even in harms [4]. For example, hydroxychloroquine gained wide attention as a possible treatment for COVID-19 due to favorable results found in in-vitro or small uncontrolled studies [5]. However, later, randomized trials in hospitalized patients, such as the RECOVERY trial [6] and the Solidarity trial [3], failed to find any clinical benefit compared to usual care. This is similar to what happened with azithromycin [7–10], while there are still few well-designed trials that have assessed other medications such as ivermectin [11–13] or vitamins supplements [14,15]."
oAlso, we added the following to the second and third paragraphs of the introduction:
Line 36-40: "This may lead to unintended consequences, such as adverse events, unnecessary expenses, delay in attending professional evaluation, masking of symptoms, and drug interactions [17–19]. Self-medication prevalence varies according to several factors, such as the methodology used to assess self-medication [20], the population characteristics [20–22], and across different countries and contexts [23–25]."
Line 41-43: "Previous studies have assessed the prevalence and characteristics of COVID-19 self-medication, reporting the use of several medications, herbal products, and dietary supplements as treatment or prevention for COVID-19 [27]. However, to date, no systematic review has summarized their findings."

Methods

•R2C4: Data source. It would be good if the authors can include the terms used for the papers search, as this is an important component for a systematic review.
•Thank you for your observation. The keywords used for the search in each database are available in the supplementary material (S1 Table). This is mentioned now in the second paragraph of the Methods:
oLine 52-53: "Search strategies were constructed a priori using different terms related to 'COVID-19' and 'self-medication' (Supplementary Table S1)."

Results

•R2C5: Definition of self-medication. If 9 of the 11 studies did not mention on the definition of self-medication, how did the author include these papers in the study? More explanation would be very useful. Please explain this in the manuscript. This showed the importance of providing the keywords for article search.
oThank you for your observation. To clarify this, we have added the following at the end of the first paragraph of the "inclusion criteria" subheading:
Line 63-65: "We considered that a study assessed the use of self-medication in any of the following situations: 1) the study reported explicitly that self-medication was assessed (regardless of the definition used), or 2) study assessed the use of medications without medical prescription."

•R2C6: Who suggested the practice of self-medication. In the results it stated that seeking advice from doctors and medical guideline are included as the source to the advice on self-medication. But if someone get an advice from a doctor, would it still be defined as self-medication? This goes back to the concern on the definition of the self-medication and the selection of papers in this study. Please explain.

Powered by Editorial Manager® and ProduXion Manager® from Aries Systems Corporation

	oThanks for the observation. We have discussed the subject, and we consider that the self-medication definition is incompatible with the situations in which participants consulted with physicians regarding their medications.<br>oAccordingly, we have added this definition in the "inclusion criteria" subsection of the methods:<br>oLine 63-65: "We considered that a study assessed the use of self-medication in any of the following situations: 1) the study reported explicitly that self-medication was assessed (regardless of the definition used), or 2) the study assessed the use of medications without medical prescription."<br>oAlso, we reviewed again all the studies, and excluded 3 of them which did not match with this self-medication definition: Ahmed (2020), Kamarli Altun (2020), and Chauhan (2020). Accordingly, the review now includes only 8 studies, and all the articles have been updated.<br><br>Discussion<br>•R2C7: It would be good if the author may emphasize on the importance of the findings and why this paper is worth to be published.<br>oWe agree with the recommendation. Accordingly, we added the following as the last paragraph of the discussion (right before the conclusion):<br>oLine 244-249: "The findings of this review suggest that there is an important though heterogeneous prevalence of self-medication to prevent or manage COVID-19. This includes some medications that have not shown any benefit so far and may therefore expose people to unnecessary adverse events. The results vary widely across studies, suggesting that each context (region, country) has its own self-medication patterns and impact, calling on local health authorities to promote research and interventions to reduce potential self-medication adverse consequences."<br><br>•R2C8: It would be good if the author may discuss on the future study or research gap related to the topic.<br>oThanks for the suggestion. We added the following as the penultimate paragraph of the discussion:<br>oLine 239-243: "Due to the limitations of the primary studies included in this review, more well-designed and adequately reported studies are needed, as well as the use of standardized self-medication definitions across studies. Moreover, since the COVID-19 status is changing rapidly, future studies are required to assess if there is any variation in self-medication trends between COVID-19 waves and after introducing the massive vaccination."<br><br><br>Reviewer #3<br><br>•R3C1: Line 5 and 6 "Suppose the study aimed to assess the frequency of self-medication for the prophylaxis or management of COVID-19. In that case, I was wondering why the authors included studies that assessed self-medication for all reasons?"<br>oWe understand the concern of the reviewer. To clarify our inclusion criteria, we have changed the redaction as follows:<br>Line 59-62: “We included original studies, either published in scientific journals or with full text available in preprint repositories, which reported original data and assessed the use of self-medication to prevent or manage COVID-19 as a primary or secondary outcome, in participants of any age and from any location.”<br>Line 63-65: “We considered that a study assessed the use of self-medication in any of the following situations: 1) the study reported explicitly that self-medication was assessed (regardless of the definition used), or 2) the study assessed the use of medications without medical prescription."<br><br><br>•R3C2: Line 12 "The authors stated that they "identified 11 studies that assessed self-medication for the prevention or management of COVID-19." It is unclear how the authors linked the self-medication in the 7 studies that assessed "self-medication for any reason" to COVID-19. The authors' definition of self-medication did not specify COVID-19, e.g., Quispe-Cañari (2021). The respondents may be self-medicating for another ailment".<br>oThank you for your observation. To clarify this, we have added the following at the

	end of the first paragraph of the "inclusion criteria" subheading:<br>Line 63-65: "We considered that a study assessed the use of self-medication in any of the following situations: 1) the study reported explicitly that self-medication was assessed (regardless of the definition used), or 2) study assessed the use of medications without medical prescription. "<br>oRegarding the study of Quispe Cañari et al, it assessed self-medication for COVID-19 prevention and management (table 2 of the results of the study, link: https://www.ncbi.nlm.nih.gov/pmc/articles/PMC7832015/). These are the results that we took into account.<br><br>•R3C3: Line 19 "It is unclear why the authors chose the term frequency instead of prevalence throughout the manuscript. 'Prevalence' may be more appropriate to use in this context".<br>oWe agree and have made such changes throughout the manuscript.<br><br>•R3C4: Line 29 "Replace 'now' with 'later'"<br>oAs we modified the writing of the manuscript, this suggestion is not valid anymore.<br><br>•R3C5: Line 33 to 35 "The sentence needs to be rephrased to reveal the intended meaning. Consider rephrasing to: "Previous studies have assessed the frequency and characteristics of COVID-19 self-medication to figure out which medications are being used that are ineffective or potentially dangerous and which factors predispose people to self-medication"<br>oThanks for the observation. We have changed the following:<br>Line 2-3: "Previous studies have assessed the prevalence and characteristics of self-medication in COVID-19. However, no systematic review has summarized their findings."<br>Line 41-43: "Previous studies have assessed the prevalence and characteristics of COVID-19 self-medication, reporting the use of several medications, herbal products, and dietary supplements as treatment or prevention for COVID-19 [26]. However, to date, no systematic review has summarized their findings."<br><br>•R3C6: Line 34 "The use of the term 'useless' is unsuitable for scientific writing. Consider using 'ineffective'"<br>oAs we modified the writing of the manuscript, this suggestion is not valid anymore.<br><br>•R3C7: Line 43 "I wondered if it is possible to search all these databases in a single day without any pre-defined search guide. It will be good to state that the search terms were developed a priori for better clarity to any reader".<br>oWe agree with the recommendation, So, in order to guide the readers, we added the following line in "Data sources" section:<br>Line 52-53: "Search strategies were constructed a priori using different terms related to 'COVID-19' and 'self-medication' (Supplementary Table S1)."<br><br>•R3C8: Line 44 to 45 "This is very nice. By not restricting, you avoid what is called "language bias". However, since no language restriction was applied, it will be good for the authors to state how articles found that are not written in English were interpreted? Assuming none was found, the interpretation criteria should have been mentioned in the protocol, but unfortunately, it was not stated either in the protocol".<br>oThanks for the observation. We added the following lines in the "Data sources" section to clarify this:<br>Line 55-56: "We planned to hire a translation service if any study written in a language other than English or Spanish was found."<br><br>•R3C9: Line 120 "The Table title does not tally with the content. It states, "…. assessed self-medication of any drug for any reason". The title will assume that studies that assessed self-medication for the prevention and treatment of COVID-19 will not be included. The title needs to be rephrased to be all-encompassing. "Characteristics and findings of studies that assessed self-medication practices during the COVID-19 pandemic""<br>oWe merged tables 2 y 3, and this new table 2 have the following title:<br>Line 131-132: "Characteristics and findings of studies that assessed self-medication to prevent or manage COVID-19."

	•R3C10: Table 3 "In the 3rd column in the 1st row, i.e., Mansuri (2020), it is unclear how you presented the Subjects (age, sex). It seems to be in percentage. The same applies to Zavala-Flores (2020). The unit of age should be stated, i.e., years, months, e.t.c."<br>oWe agree and have made the change, in the first row of the third column of table 3 now it says "age: 60.8% were less than 40 years, female: 60.3%". Then, in the last row of the third column of table 2, now it says, "Mean age: 60.3% years".<br><br>•R3C11: Line 174 "Kindly rephrase to "Only one study [22] specified the source of the patients' self-medications""<br>oWe agree and have made such change in the results. Please, see the second paragraph (line 180) of the "Reasons to practice self-medication, from where were the medications obtained, and adverse effects" subheading in the results.<br><br>•R3C12: Line 203 "Current guidelines??? It will be good to mention which guideline the authors are referring to. COVID-19 guideline?? By which body?? WHO, CDC etc"<br>oWe consider the recommendation, so we rephrased the sentence (4th paragraph of the discussion). Now it says:<br>Line 215-216: "As stated by the current COVID-19 guidelines by WHO and IDSA [41,42]."<br><br>•R3C13: Throughout the manuscript "The manuscript needs detailed proofreading and revision for its English"<br>oWe agree with the recommendation, and a translator has checked the revised version of this manuscript.<br><br>Reviewer #4<br><br>•R4C1: Page 3, paragraph 3: "…we aimed to assess the frequency of self-medication…". Please explain the reason of using the term "frequency" rather than "prevalence".<br>oWe have reviewed the differences between the two terms and agree with your suggestion. So that, we replaced 'frequency' by 'prevalence' throughout the manuscript.<br><br>•R4C2: Page 5-6, Table 1: Both the final score for Ahmed (2020) and Chauhan (2020) are zero. Please explain the rational of include these two articles in the systematic review although the scores are zero.<br>oDue to the commentaries of another reviewer, we have realized that 3 of the initially included studies did not fulfil the self-medication definition because they included medication taken due to a prescription. We have excluded these 3 studies from the results: Ahmed (2020), Kamarli Altun (2020), and Chauhan (2020).<br><br>•R4C3: Page 8, paragraph 2: "Of these seven studies, only two specified which question was asked, whereas the other five studies did not detail whether participants could report self-medication only for a prespecified list of drugs or whether it was an open question". The used of a prespecified list of drugs might narrow down the answers from the respondents. This subsequently might lead to biases in the study findings. This issue needs to be discussed in the discussion section.<br>oWe agree with your suggestion and decide to add the following sentence (6th paragraph of the discussion):<br>Line 232-234: "In addition, it is possible that studies that evaluated self-medication through a pre-specified list of medications reported biased prevalences, since these medications may not have included all the most commonly used medications [47]."<br><br>47.- Choi B, Granero R, Pak A. Catalog of bias in health questionnaires. Rev Costarr Salud Pública. 2010;19: 106–118. Available: http://www.scielo.sa.cr/scielo.php?script=sci_arttext&pid=S1409-14292010000200008&lng=en.
**Additional Information:**	
**Question**	**Response**

Powered by Editorial Manager® and ProduXion Manager® from Aries Systems Corporation

**Financial Disclosure**<br><br>Enter a financial disclosure statement that describes the sources of funding for the work included in this submission. Review the submission guidelines for detailed requirements. View published research articles from *PLOS ONE* for specific examples.<br><br>This statement is required for submission and **will appear in the published article** if the submission is accepted. Please make sure it is accurate.<br><br>**Unfunded studies**<br>Enter: *The author(s) received no specific funding for this work.*<br><br>**Funded studies**<br>Enter a statement with the following details:<br>• Initials of the authors who received each award<br>• Grant numbers awarded to each author<br>• The full name of each funder<br>• URL of each funder website<br>• Did the sponsors or funders play any role in the study design, data collection and analysis, decision to publish, or preparation of the manuscript?<br>• **NO** - Include this sentence at the end of your statement: *The funders had no role in study design, data collection and analysis, decision to publish, or preparation of the manuscript.*<br>• **YES** - Specify the role(s) played.<br><br>* typeset	The author(s) received no specific funding for this work.
**Competing Interests**<br><br>Use the instructions below to enter a competing interest statement for this submission. On behalf of all authors, disclose any competing interests that could be perceived to bias this work—acknowledging all financial support and any other relevant financial or non-financial competing interests.<br><br>This statement is required for submission	The authors have declared that no competing interests exist.

and **will appear in the published article** if the submission is accepted. Please make sure it is accurate and that any funding sources listed in your Funding Information later in the submission form are also declared in your Financial Disclosure statement.<br><br>View published research articles from *PLOS ONE* for specific examples.<br><br>**NO authors have competing interests**<br><br>Enter: *The authors have declared that no competing interests exist.*<br><br>**Authors with competing interests**<br><br>Enter competing interest details beginning with this statement:<br><br>*I have read the journal's policy and the authors of this manuscript have the following competing interests: [insert competing interests here]*<br><br>* typeset	
**Ethics Statement**<br><br>Enter an ethics statement for this submission. This statement is required if the study involved:<br><br>• Human participants<br>• Human specimens or tissue<br>• Vertebrate animals or cephalopods<br>• Vertebrate embryos or tissues<br>• Field research<br><br>Write "N/A" if the submission does not require an ethics statement.<br><br>General guidance is provided below. Consult the submission guidelines for detailed instructions. **Make sure that all information entered here is included in the Methods section of the manuscript.**	N/A

**Format for specific study types**<br><br>**Human Subject Research (involving human participants and/or tissue)**<br>• Give the name of the institutional review board or ethics committee that approved the study<br>• Include the approval number and/or a statement indicating approval of this research<br>• Indicate the form of consent obtained (written/oral) or the reason that consent was not obtained (e.g. the data were analyzed anonymously)<br><br>**Animal Research (involving vertebrate animals, embryos or tissues)**<br>• Provide the name of the Institutional Animal Care and Use Committee (IACUC) or other relevant ethics board that reviewed the study protocol, and indicate whether they approved this research or granted a formal waiver of ethical approval<br>• Include an approval number if one was obtained<br>• If the study involved *non-human primates*, add *additional details* about animal welfare and steps taken to ameliorate suffering<br>• If anesthesia, euthanasia, or any kind of animal sacrifice is part of the study, include briefly which substances and/or methods were applied<br><br>**Field Research**<br><br>Include the following details if this study involves the collection of plant, animal, or other materials from a natural setting:<br>• Field permit number<br>• Name of the institution or relevant body that granted permission	
**Data Availability**<br><br>Authors are required to make all data underlying the findings described fully available, without restriction, and from the time of publication. PLOS allows rare exceptions to address legal and ethical concerns. See the PLOS Data Policy and FAQ for detailed information.	Yes - all data are fully available without restriction

Powered by Editorial Manager® and ProduXion Manager® from Aries Systems Corporation

A Data Availability Statement describing where the data can be found is required at submission. Your answers to this question constitute the Data Availability Statement and **will be published in the article**, if accepted.<br><br>**Important:** Stating 'data available on request from the author' is not sufficient. If your data are only available upon request, select 'No' for the first question and explain your exceptional situation in the text box.<br><br>Do the authors confirm that all data underlying the findings described in their manuscript are fully available without restriction?	
**Describe where the data may be found in full sentences. If you are copying our sample text, replace any instances of XXX with the appropriate details.**<br><br>• If the data are **held or will be held in a public repository**, include URLs, accession numbers or DOIs. If this information will only be available after acceptance, indicate this by ticking the box below. For example: *All XXX files are available from the XXX database (accession number(s) XXX, XXX.*).<br>• If the data are all contained **within the manuscript and/or Supporting Information files**, enter the following: *All relevant data are within the manuscript and its Supporting Information files.*<br>• If neither of these applies but you are able to provide **details of access elsewhere**, with or without limitations, please do so. For example:<br><br>*Data cannot be shared publicly because of [XXX]. Data are available from the XXX Institutional Data Access / Ethics Committee (contact via XXX) for researchers who meet the criteria for access to confidential data.*<br><br>*The data underlying the results presented in the study are available from (include the name of the third party*	All relevant data are within the paper and its Supporting Information files.

and contact information or URL).<br>• This text is appropriate if the data are owned by a third party and authors do not have permission to share the data.<br><br>* typeset	
Additional data availability information:	Tick here if your circumstances are not covered by the questions above and you need the journal’s help to make your data available.

Powered by Editorial Manager® and ProduXion Manager® from Aries Systems Corporation

Manuscript Click here to access/download;Manuscript;Manuscript.docx

Self-medication practices to prevent or manage COVID-19: a systematic review

Short title: Self-medication practices for COVID-19

Authors:

Alvaro Quincho-Lopez[1]

Christeam A. Benites-Ibarra[2]

Maryori M. Hilario-Gomez[1]

Renatta Quijano-Escate[3]

Alvaro Taype-Rondan[4]

Affiliations:

[1]Universidad Nacional Mayor de San Marcos, Sociedad Científica de San Fernando, Lima, Peru

[2]Sociedad Científica de Estudiantes de Medicina de la Universidad Nacional del Santa, Nuevo Chimbote, Ancash, Peru

[3]Universidad Nacional San Luis Gonzaga, Sociedad Científica de Estudiantes de Medicina de Ica, Ica, Peru

[4]Universidad San Ignacio de Loyola, Unidad de Investigación para la Generación y Síntesis de Evidencias en Salud, Lima, Peru

Funding:

This study was self-funded by the authors.

Corresponding author:

Alvaro Taype-Rondan

Universidad San Ignacio de Loyola, Unidad de Investigación para la Generación y Síntesis de Evidencias en Salud

Av. La Fontana 750, Lima, Peru

alvaro.taype.r@gmail.com

Abstract

Background: Previous studies have assessed the prevalence and characteristics of self-medication in COVID-19. However, no systematic review has summarized their findings.

Objective: We conducted a systematic review to assess the prevalence of self-medication to prevent or manage COVID-19.

Methods: We used different keywords and searched studies published in PubMed, Scopus, Web of Science, Embase, two preprint repositories, Google, and Google Scholar. We included studies that reported original data and assessed self-medication to prevent or manage COVID-19. The risk of bias was assessed using the Newcastle–Ottawa Scale (NOS) modified for cross-sectional studies.

Results: We identified eight studies, all studies were cross-sectional, and only one detailed the question used to assess self-medication. The recall period was heterogeneous across studies. Of the eight studies, seven assessed self-medication without focusing on a specific symptom: four performed in the general population (self-medication prevalence ranged between <4% - 88.3%) and three in specific populations (range: 33.9% to 51.3%). In these seven studies, the most used medications varied widely, including antibiotics, chloroquine or hydroxychloroquine, acetaminophen, vitamins or supplements, ivermectin, and ibuprofen. The last study only assessed self-medication for fever due to COVID-19. Most studies had a risk of bias in the "representativeness of the sample" and "assessment of outcome" items of the NOS.

Conclusions: Studies that assessed self-medication for COVID-19 found heterogeneous results regarding self-medication prevalence and medications used. More well-designed and adequately reported studies are warranted to assess this topic.

Keywords: COVID-19, SARS-CoV-2, Self-Medication, Global Health, Pharmaceutical Preparations, Systematic Review

1 Introduction

With the progression of the COVID-19 pandemic, several medications have been proposed as potential candidates for this disease [1], most of which resulted in little or no benefit for the patients [2,3] or even in harms [4]. For example, hydroxychloroquine gained wide attention as a possible treatment for COVID-19 due to favorable results found in in-vitro or small uncontrolled studies [5]. However, later, randomized trials in hospitalized patients, such as the RECOVERY trial [6] and the Solidarity trial [3], failed to find any clinical benefit compared to usual care. This is similar to what happened with azithromycin [7–10], while there are still few well-designed trials that have assessed other medications such as ivermectin [11–13] or vitamins supplements [14,15].

Nonetheless, the fear of contracting the virus, low access to health services, and massive misinformation have led some people to self-medicate. According to the World Health Organization (WHO), self-medication "involves the use of medicinal products by the consumer to treat self-recognized disorders or symptoms." [16]. This may lead to unintended consequences, such as adverse events, unnecessary expenses, delay in attending professional evaluation, masking of symptoms, and drug interactions [17–19]. Self-medication prevalence varies according to several factors, such as the methodology used to assess self-medication [20], the population characteristics [20–22], and across different countries and contexts [23–25].

Previous studies have assessed the prevalence and characteristics of COVID-19 self-medication, reporting the use of several medications, herbal products, and dietary supplements as treatment or prevention for COVID-19 [26]. However, to date, no systematic review has summarized their findings.

Thus, in this systematic review, we aimed to assess the prevalence of self-medication for the prevention or management of COVID-19. In addition, we assessed the type of medication used, reasons to practice self-medication, from where such medications were obtained, and adverse events due to its practice.

2 Methods

This systematic review was reported according to the Preferred Reporting Items for Systematic Reviews and Meta-Analyses (PRISMA) guidelines (Supplementary Table S3) [27]. The study protocol has been registered at PROSPERO (CRD42021236191).

2.1 Data sources

Search strategies were constructed a priori using different terms related to 'COVID-19' and 'self-medication' (Supplementary Table S1). On February 4th, 2021, we searched PubMed, Scopus, Web of Science, Embase, two preprint repositories (MedRxiv and SciELO Preprints), and grey literature sources (Google and Google Scholar). No language restriction was applied. We pla[illegible]l to hire a translation service if any study written in a language other than English or Spanish was found. The search was restricted to include documents published in 2020 or 2021.

2.2 Inclusion criteria

We included original studies, either published in scientific journals or with full text available in preprint repositories, which reported original data and assessed the use of self-medication to prevent or manage COVID-19 as a primary or secondary outcome, in participants of any age and from any location.

We considered that a study assessed the use of self-medication in any of the following situations: 1) the study reported explicitly that self-medication was assessed (regardless of the definition used), or 2) the study assessed the use of medications without medical prescription.

2.3 Study selection

Two authors (AQL and MHG) independently selected the studies. For this, they first screened the search results by title and abstract according to the inclusion criteria using the web-based tool Rayyan (http://rayyan.qcri.org). Later, they reviewed the full text of the relevant studies to determine whether they were appropriate for study inclusion. Discrepancies were consulted with another author (ATR) and resolved by consensus.

2.4 Data extraction

Two authors (CBI and RQE) independently extracted the relevant data for the review using a pre-piloted Microsoft Excel spreadsheet. Again, any discrepancies were discussed with another author (ATR) and resolved with consensus.

The following variables were ~~assessed~~ for each study: author, year of publication, country, study design, setting, population, sample technique, date of data collection, number of participants, age, sex, how was self-medication assessed, the prevalence of self-medication, reasons for practicing self-medication, who recommended such medication, from where was the medication obtained, and adverse events related to its use.

2.5 Risk of bias assessment

Two authors (CBI and RQE) independently evaluated the risk of bias for each included study using the Newcastle–Ottawa Scale (NOS) adapted for descriptive cross-sectional studies [28]. This scale comprises two criteria: the selection criteria (representativeness of the sample, sample size, and nonresponders) and the outcome criteria (outcome assessment and statistical tests). Any discrepancy was discussed with another author (ATR).

2.6 Statistical analysis

Because studies were performed in different populations and using different definitions of self-medication, we did not perform meta-analyses and decided to present the results of each study separately.

3 Results

3.1 Study selection

A total of 765 records were found in the database search. After the duplicates were eliminated, 227 records were screened, of which 46 documents were assessed in full-text. Lastly, 38 papers were

excluded (which are detailed in Supplementary Table S2), and 8 papers were included, all of which were cross-sectional studies (Fig. 1).

[Insert Figure 1]

Fig 1. Flow diagram of study selection.

3.2 Methodological quality of the included studies

We assessed the risk of bias using the NOS. The study performed by Sadio [29] included two samples: one obtained using a probabilistic sample, and the other used a convenience sample. Thus, we assessed the risk of bias of these two samples separately.

Of the eight studies, only one (Sadio probabilistic sample) fulfilled the five stars in the NOS scale. In addition, the Sadio non-probabilistic sample, along with the other three studies, received four out of five stars. All studies except one (Sadio probabilistic sample) failed in ~~representing~~ the sample item of the ~~selection~~ criteria (Table 1).

Table 1. Risk of bias assessment of included studies using the Newcastle–Ottawa Scale adapted for cross-sectional studies.

Study: Author (year)	Final score	Representativeness of the sample[1]	Sample size[2]	Not respondents[3]	Outcome evaluation[4]	Statistical test[5]
Dare (2021)	2/5			★		★
Nasir (2020)	1/5				★	
Quispe-Cañari (2021)	4/5		★	★	★	★
Wegbom (2021)	3/5		★	★		★
Miñan-Tapia (2020)	4/5		★	★	★	★
Sadio (2021)[6]	5/5	★	★	★	★	★
Sadio (2021)[7]	4/5		★	★	★	★
Zavala-Flores (2020)	1/5				★	
Mansuri (2020)	4/5		★	★	★	★

[1]Representativeness of the sample: One star was given to studies with random sampling or census.
[2]Sample size: One star was given to studies with justified and satisfactory sample size.
[3]Non-respondents: If comparability between respondents' and nonrespondents' characteristics was established and the response rate was satisfactory, one star was given.
[4]Outcome evaluation: If the study explicitly mentioned how self-medication was defined and how long the recall period was, one star was given.
[5]Statistical test: One star was given if it did not use a complex sample and the sample had been calculated correctly, or if it used a complex sample and such sampling was considered to estimate the self-medication prevalence.
[6]Probability sampling.
[7]Nonprobability sampling.

3.3 Studies characteristics

Studies characteristics are shown in Table 2. The eight included studies reported data on self-medication practices in six different countries: three from Peru [30–32], one from Bangladesh [33], one from Togo [29], one from Nigeria [34], one from Uganda [35], and one from the Kingdom of Saudi Arabia [36]. All studies were cross-sectional. Regarding their publication, five studies were published as original reports [29,30,33,34,36], one was a letter to the editor [31], and two were preprints reporting original data [32,35]. Studies were performed from March to August 2020.

Table 2. Characteristics and findings of studies that assessed self-medication to prevent or manage COVID-19.

**Study: Author (year)**	**Country (city) / date of study**	**Subjects (age, sex)**	**Survey application and self-medication definition**	**Prevalence of self-medication for COVID-19 prevention or management (overall and most-used medications)**
**Studies that assessed self-medication without focusing on a specific symptom, performed in the general population:**				
Dare (2021) - preprint	Uganda (Western region) / from June to August 2020	Adults from Uganda western cities (age: 43.8% between 25 and 34 years, female: 45.2%), N = 272	• In-person and online survey (convenience sampling).<br>• Self-medication assessment was not detailed.<br>• Recall period: during the COVID-19 pandemic.	• **Overall:** 57%.
Nasir (2020)	Bangladesh (Dhaka city) / from April to June 2020	Adults (age: 50.5% between 45 and 54 years, female: 55.3%), N = 626	• Open online survey (convenience sampling).<br>• Self-medication was defined as taking medications without prescription.<br>• Recall period: during the COVID-19 outbreak.	• **Overall:** 88.3%.<br>• Ivermectin (77.2%), azithromycin (54.2%), montelukast (43.1%), calcium supplements (41.4%), doxycycline (40.3%), hydroxychloroquine (20.4%).
Quispe-Cañari (2021)	Peru / June 2020	Adults (median age: 23 years, female: 54.5%), N = 3792	• Open online survey (convenience sampling).<br>• Respondents were asked to indicate whether they consumed any of the following medications: acetaminophen, ibuprofen, azithromycin, hydroxychloroquine, penicillin, or antiretrovirals.<br>• Recall period: during the COVID-19 lockdown.	• **Overall:** not reported, but taking into account the prevalence for each medication, overall prevalence was < 4%.<br>• **For prevention:** azithromycin (0.2%), acetaminophen (0.2%), hydroxychloroquine (0.1%), antiretrovirals (0.1%), penicillin (0.1%) ibuprofen (0.03%).<br>• **For management:** acetaminophen (1.7%), azithromycin (0.6%), ibuprofen (0.2%), antiretrovirals (0.1%), hydroxychloroquine (0.03%), penicillin (0.03%).
Wegbom (2021)	Nigeria / from June to July 2020	Adults (mean age: 42.2 years, female: 57.1%), N = 461	• Open online survey (convenience sampling).<br>• Self-medication was defined as taking medications for the prevention or management of COVID-19 without prescription by medically qualified personnel.<br>• Recall period: not detailed.	• **Overall:** 41%.<br>• Vitamin C or multivitamin (21.3%), antimalarial drugs other than hydroxychloroquine and chloroquine (19.3%), amoxicillin (10.2%), ciprofloxacin (6.1%), herbal products (4.1%), metronidazole (3.5%), erythromycin (2.2%), hydroxychloroquine or chloroquine (1.3%).
**Studies that assessed self-medication without focusing on a specific symptom, performed in a specific population:**				
Miñan-Tapia (2020) - preprint	Peru (Tacna) / from June to July 2020	Undergraduate students of health-related careers from two universities (median age: 21 years, female: 69.1%), N = 718	• Online, convenience sampling.<br>• Self-medication was defined as using any of the pre-specified 14 medicines for COVID-19 without medical prescription.<br>• Recall period: during the past three months.	• **Overall:** 51.3%<br>• Acetaminophen (21.2%), ibuprofen (10.8%), dexamethasone (5.5%), aspirin (4.4%), azithromycin (2.5%).

Sadio (2021)	Togo (Lomé) / from April to May 2020	Adults from any of these five sectors: health care, air transport, police, road transport, and informal (median age: 36 years, female: 28.4%), N = 955	• In-person survey through recruitment, open invitation, and random sampling (two or three stages).<br>• Self-medication was defined as the use of drugs alleged to treat or prevent COVID-19 without a physician order.<br>• Recall period: 2 weeks.	• **Overall:** 34.2%<br>• Vitamin C (27.6%), traditional medicine (10.2%), chloroquine/hydroxychloroquine (2.0%), azithromycin (1.2%).
Zavala-Flores (2020) – letter to the editor	Peru (Lima) / June 2020	Hospitalized adults with COVID-19 (Mean age: 60.3 years, Female: 30.3%), N = 132	• The authors included all patients hospitalized during a specific week.<br>• Self-medication was defined as using some COVID-19 medication before hospitalization without medical prescription.<br>• Recall period: since the COVID-19 diagnosis.	• **Overall (for COVID-19 management):** 33.9%<br>• Antibiotics (28.3%), ivermectin (20.7%), corticosteroids (17.0%), acetaminophen (12.3%), aspirin (4.7%), NSAID 84.7%), hydroxychloroquine (0.9%), others (1.9%).
**Studies that assessed the use of medications for a specific symptom:**				
Mansuri (2020)	Kingdom of Saudi Arabia / from March to April 2020	Adults (age: 60.8% were less than 40 years, female: 60.3%), N = 388	• An online electronic survey, convenience sampling.<br>• Self-medication was assessed using the question, "Are you self-medicating for fever?"<br>• Recall period: during the COVID-19 pandemic.	• **Overall:** 35.1% to manage fever.

NSAID, nonsteroidal anti-inflammatory drug

Regarding the population, five studies were conducted targeting the general population, one was conducted in hospitalized patients with COVID-19 [31], one in healthcare undergraduate students [32], and one in workers from five sectors (health care, air transport, police, road transport, and informal) [29].

The eight studies included between 132 and 3,792 participants. Five studies mentioned the summary measure (mean or median) of the participants' age. Such summary measure (either mean or median) ranged from 21 to 60.3 years. All studies were performed in adults. The percentage of females ranged from 28.4% to 69.1% (Table 2).

3.4 Definition of self-medication

All studies assessed self-medication through self-report. The questions used to assess self-medication were explicitly mentioned only in the one study [36], while other five studies report having asked for the use of medicine without prescription [29,31–34], and two studies gave no details regarding how self-medication was measured in their surveys [30,35]. Moreover, three of the eight studies [30,32,36] reported collecting only a pre-specified list of medications.

Regarding the recall period for which self-medication was assessed: three studies assessed it during the COVID-19 pandemic [33,35,36], one during the COVID-19 lockdown [30], one during the past three months [32], one during the past two weeks [29], one since the COVID-19 diagnosis [31], and one did not detail the recall period [34] (Table 2).

3.5 Prevalence of self-medication and most frequently used medications

As showed in Table 2, We classified the eight included studies into three groups: 1) those that assessed self-medication for COVID-19 prevention or management without focusing on a specific symptom, and 2) those that assessed self-medication for a specific COVID-19 symptom.

Seven studies were included in the first group (assessed self-medication for COVID-19 prevention or management without focusing on a specific symptom). Of these, four were performed in the general population (with a range of self-medication prevalence between <4% and 88.3%) and three in specific populations (which reported a self-medication prevalence of 33.9% for hospitalized adults with COVID-19, 34.2% in adults who worked in five assessed sectors, and 51.3% in undergraduate students of health-related careers).

Of these seven studies, six mentioned the list of medications reported by the participants. The reported medications were different across the studies: six studies reported the consumption of antibiotics, five of chloroquine or hydroxychloroquine, three of acetaminophen, three of vitamins or supplements, two of ivermectin, and two of ibuprofen.

In addition, consumption preferences varied across studies. For example, when medications were sorted in the order of highest to lowest consumption, vitamins/supplements occupied the first place in two studies, but the fourth place in another study (Table 2).

The second group (studies that assessed the use of medication for a specific symptom) included one study performed on the general population of Saudi Arabia, which found that 35.1% of the surveyed individuals used self-medication for fever [36] (Table 2).

3.6 Reasons to practice self-medication, from where were the medications obtained, and adverse effects

Four of the eight included studies mentioned the reasons for self-medication. However, each study assessed the reasons differently. Thus, one study referred only to the symptoms that motivated participants to self-medicate, and the other three collected a plethora of reasons, including the fear of stigmatization, fear of quarantine, affordability, the convenience of self-medication, and patients believing that the symptoms were not severe (Table 3).

Only one study [34] specified the source of the patients' self-medications. This study was performed on adults from Nigeria. The most common sources reported were the pharmacy (73.9%) and the patent medicine vendor (23.6%).

Only two studies reported whether adverse events occurred in those who self-medicate. One study performed on Undergraduate students of health-related careers found that 11.7% of those who self-medicate presented with side effects of self-medication in the past three months [32]. Nonetheless, this study did not specify the adverse effects that occurred. The second study, performed in adults from Nigeria, reported body rash (23.1%) followed by worsened condition (17.3%), yellowish eyes (7.7%), swollen face (3.8%), and vomiting of blood and severe diarrhea (5.8%) [34].

Table 3. Reasons to practice self-medication, from where were the medications obtained, and adverse effects

Study: Author (year)	Reasons to practice self-medication	Obtained from	Adverse effects
Nasir (2020)	Fever (37.6%), throat pain (28.8%), dry cough (14.2%), loss of smell (9.2%), loss of taste (3.5%), body ache (5.0%), and rarely diarrhea (1.7%)	NR	NR
Wegbom (2021)	Fear of stigmatization or discrimination (79.5%), fear of quarantine or self-isolation (77.3%), fear of infection or contact with a suspected or known COVID-19 patient (76.3%), delay in receiving treatment at health facilities" (55.6%), influence of friends" (55.2%), social media (54.3%), nonavailability of medications for COVID-19 treatment in the health facilities (53%), emergency illness (49.1%), delay in getting hospital services (28.1%), distance to the health facility (23%), proximity of the pharmacy (21%), nonavailability of medicine in a health facility (19.3%), and health facility charges (15.3%)	Pharmacy (73.9%), patent medicine vendor (23.6%), hospital (7.6%), hawkers (4.5%), faith-based outlets, and herbalists (2.1%, each)	Body rash (23.1%) followed by worsened condition (17.3%), yellowish eyes (7.7%), swollen face (3.8%), and vomiting of blood and severe diarrhea (5.8%).
Dare (2021)	Self-medication is affordable (37%) and convenient (32%), lack the means to get to the health	NR	NR

	facility/hospital (15%), fear of being diagnosed COVID-19 positive (9%), fear of visiting health facility or hospital (7%)		
Miñan-Tapia (2020)	Believing that the symptoms were not severe enough to go to a doctor (64.3%), referring to family/friends that are nonmedical health professionals (34.9%), and owing to economic reasons or use of over-the-counter medications (34.9%)	NR	11.7% participants reported adverse events (not detailed)

NR: not reported

Note: The percentage was only based on the people who self-medicated in each study.

4 Discussion

To our knowledge, this is the first systematic review to assess the self-medication prevalence for COVID-19 prevention or management. It assessed the most important databases and sources for grey literature search and preprints repositories; without language limitations. Thus, our results should reflect the state of knowledge until the search date.

Self-medication is a global phenomenon that may involve health risks at both the individual and community levels [19,37]. Previous studies have found that self-medication is a common practice. Three systematic reviews conducted in Iran [23], Ethiopia [25], and India [24]; reported a prevalence of 53%, 44%, and 53.6%, respectively; for a recall period that ranged from a single day to 6 months.

In our review, we only found eight studies. Although the included studies were performed in six countries, the lack of studies in some regions such as North America, the Middle East, North Africa, and Oceania is evident. It is expected that the intercountry differences in drug promotion, regulations, and the possibility of accessing some medications without a prescription can influence the self-medication patterns [38,39]. Moreover, seven of the eight included studies were performed in low- and middle-income countries. While self-medication may be higher in such countries, due to the conditions and structure of the health system [40], it is not possible to make solid comparisons due to the heterogeneity in the self-medication assessment across studies.

Some of the included studies assessed over-the-counter medications such as acetaminophen or nonsteroidal anti-inflammatory drugs, which are used for the symptom management of COVID-19, along with several other diseases. However, the included studies have also found an heterogeneous

prevalence of self-medication with medications that have not proven to benefit in the prevention or management of COVID-19 (such as antibiotics, chloroquine or hydroxychloroquine, vitamins or supplements, ivermectin, and antiasthmatics), as stated by the current COVID-19 guidelines by WHO and IDSA [41,42].

In fact, the adverse effects of some of these medications are of great concern, such as antibiotic resistance due to extensive antibiotic use, bleeding caused by aspirin use, inhibition of the immune system caused by corticosteroids, or arrhythmia caused by hydroxychloroquine [4]. Some healthcare organizations have issued statements on self-medication. For instance, the WHO recognizes that a "successful" (i.e., beneficial) self-medication can be achieved in many countries only by improving individuals' knowledge and education level in such a way that the potential damages of this practice can be avoided [16]. Similarly, the International Pharmaceutical Federation, in tandem with the World Self-Medication Industry [43], and the World Medical Association [44], emphasizes the responsible use of non-prescription medications.

Similar to that found in previous systematic reviews [20,21], the included studies in our review tended to use different questions to assess self-medication, or they did not specify the question used. Moreover, studies have established different recall periods, and even those that assessed self-medication since the beginning of the pandemic would have different time frames depending on the month in which they were performed. This lack of clear information prevents direct comparisons between studies and the subsequent meta-analysis of their results [45], adding a potential source of bias that affects the internal validity of the results [45,46]. In addition, it is possible that studies that evaluated self-medication through a pre-specified list of medications reported biased prevalences, since these medications may not have included all the most commonly used medications [47].

The included studies had an important risk of bias, mainly in the "representativeness of the sample" domain of the NOS, the worst-rated item in the risk of bias assessment. This prevents the adequate extrapolation of the results [48], which should be taken into account when interpreting the results of this review.

Due to the limitations of the primary studies included in this review, more well-designed and adequately reported studies are needed, as well as the use of standardized self-medication definitions across studies. Moreover, since the COVID-19 status is changing rapidly, future studies are required to assess if there is any variation in self-medication trends between COVID-19 waves and after introducing the massive vaccination.

The findings of this review suggest that there is an important though heterogeneous prevalence of self-medication to prevent or manage COVID-19. This includes some medications that have not shown any benefit so far and may therefore expose people to unnecessary adverse events. The results vary widely across studies, suggesting that each context (region, country) has its own self-medication patterns and impact, calling on local health authorities to promote research and interventions to reduce potential self-medication adverse consequences.

5 Conclusion

We identified eight studies that assessed the use of self-medication for the prevention or management of COVID-19. ~~Only one detailed the question used to assess self-medication.~~ The recall period was heterogeneous across studies. ~~With respect to the seven studies that assessed self-medication without focusing on a specific symptom: five performed in the general population (self-medication prevalence ranged between <4% - 88.3%) and three in specific populations~~. In these studies, the ~~most~~ used medications varied widely. More well-designed and adequately reported studies are warranted in this regard.

Acknowledgments

None.

References

1. Sanders JM, Monogue ML, Jodlowski TZ, Cutrell JB. Pharmacologic Treatments for Coronavirus Disease 2019 (COVID-19): A Review. JAMA - J Am Med Assoc. 2020;323:

1824–1836.

2. Lam S, Lombardi A, Ouanounou A. COVID-19: A review of the proposed pharmacological treatments. Eur J Pharmacol. 2020;886: 173451.

3. WHO Solidarity Trial Consortium. Repurposed Antiviral Drugs for Covid-19 — Interim WHO Solidarity Trial Results. N Engl J Med. 2021;384: 497–511.

4. Kim MS, An MH, Kim WJ, Hwang TH. Comparative efficacy and safety of pharmacological interventions for the treatment of COVID-19: A systematic review and network meta-analysis. PLoS Med. 2020;17: 1–28.

5. Ng TSB, Leblanc K, Yeung DF, Tsang TSM. Medication use during COVID-19. Can Fam Physician. 2021;67: 171–179.

6. RECOVERY Collaborative Group, Horby P, Mafham M, Al. E. Effect of Hydroxychloroquine in Hospitalized Patients with Covid-19. N Engl J Med. 2020;383: 2030–2040.

7. Furtado R, Berwanger O, Fonseca H, Al. E. Azithromycin in addition to standard of care versus standard of care alone in the treatment of patients admitted to the hospital with severe COVID-19 in Brazil (COALITION II): a randomised clinical trial. Lancet. 2020;396: 959–967.

8. Hinks TSC, Cureton L, Knight R, Wang A, Cane JL, Barber VS, et al. Azithromycin versus standard care in patients with mild-to-moderate COVID-19 (ATOMIC2): an open-label, randomised trial. Lancet Respir Med. 2021;19: 1–11.

9. Oldenburg CE, Pinsky BA, Brogdon J, Chen C, Ruder K, Zhong L, et al. Effect of Oral Azithromycin vs Placebo on COVID-19 Symptoms in Outpatients with SARS-CoV-2 Infection: A Randomized Clinical Trial. JAMA - J Am Med Assoc. 2021;326: 490–498.

10. PRINCIPLE Trial Collaborative Group, Butler CC, Dorward J, Yu LM, Gbinigie O, Hayward G, et al. Azithromycin for community treatment of suspected COVID-19 in people at increased risk of an adverse clinical course in the UK (PRINCIPLE): a randomised, controlled, open-label, adaptive platform trial. Lancet. 2021;397: 1063–1074.

11. Bartoszko JJ, Siemieniuk RAC, Kum E, Qasim A, Zeraatkar D, Ge L, et al. Prophylaxis against covid-19: Living systematic review and network meta-analysis. BMJ. 2021;373: 1–12.

12. López-Medina E, López P, Hurtado IC, Dávalos DM, Ramirez O, Martínez E, et al. Effect of Ivermectin on Time to Resolution of Symptoms Among Adults With Mild COVID-19: A

Randomized Clinical Trial. Jama. 2021; 1–10. doi:10.1001/jama.2021.3071

13. Vallejos J, Zoni R, Bangher M, Villamandos S, Bobadilla A, Plano F, et al. Ivermectin to prevent hospitalizations in patients with COVID-19 (IVERCOR-COVID19) a randomized, double-blind, placebo-controlled trial. BMC Infect Dis. 2021;21: 635.

14. Thomas S, Patel D, Bittel B, Wolski K, Wang Q, Kumar A, et al. Effect of High-Dose Zinc and Ascorbic Acid Supplementation vs Usual Care on Symptom Length and Reduction among Ambulatory Patients with SARS-CoV-2 Infection: The COVID A to Z Randomized Clinical Trial. JAMA Netw Open. 2021;4: 2–11.

15. Murai IH, Fernandes AL, Sales LP, Pinto AJ, Goessler KF, Duran CSC, et al. Effect of a Single High Dose of Vitamin D3on Hospital Length of Stay in Patients with Moderate to Severe COVID-19: A Randomized Clinical Trial. JAMA - J Am Med Assoc. 2021;325: 1053–1060.

16. World Health Organization. Guidelines for the regulatory assessment of medicinal products for use in self-medication. 2000 [cited 10 Apr 2021]. Available: https://apps.who.int/iris/handle/10665/66154

17. Tasnim S, Hossain M, Mazumder H. Impact of rumors and misinformation on COVID-19 in Social Media. J Prev Med Public Heal. 2020;53: 171–174. doi:10.3961/JPMPH.20.094

18. Onchonga D, Omwoyo J, Nyamamba D. Assessing the prevalence of self-medication among healthcare workers before and during the 2019 SARS-CoV-2 (COVID-19) pandemic in Kenya. Saudi Pharm J. 2020;28: 1149–1154.

19. Hughes CM, Mcelnay JC, Fleming GF. The benefits and risks of self-medication. Drug Saf. 2001;14: 1027–1037.

20. Shehnaz SI, Agarwal AK, Khan N. A systematic review of self-medication practices among adolescents. J Adolesc Heal. 2014;55: 467–483.

21. Jerez-Roig J, Medeiros LFB, Silva VAB, Bezerra CLPAM, Cavalcante LAR, Piuvezam G, et al. Prevalence of Self-Medication and Associated Factors in an Elderly Population: A Systematic Review. Drugs and Aging. 2014;31: 883–896.

22. Behzadifar M, Behzadifar M, Aryankhesal A, Ravaghi H, Baradaran HR, Sajadi HS, et al. Prevalence of self-medication in university students: Systematic review and meta-analysis. East Mediterr Heal J. 2020;26: 846–857.

23. Azami-Aghdash S, Mohseni M, Etemadi M, Royani S, Moosavi A, Nakhaee M. Prevalence and cause of self-medication in Iran: A systematic review and meta-analysis article. Iran J

Public Health. 2015;44: 1580–1593.

24. Rashid M, Chhabra M, Kashyap A, Undela K, Gudi SK. Prevalence and Predictors of Self-Medication Practices in India: A Systematic Literature Review and Meta-Analysis. Curr Clin Pharmacol. 2019;15: 90–101.

25. Sisay M, Mengistu G, Edessa D. Epidemiology of self-medication in Ethiopia: A systematic review and meta-analysis of observational studies. BMC Pharmacol Toxicol. 2018;19: 56.

26. Yáñez JA, Chung SA, Román BR, Hernández-Yépez PJ, Garcia-Solorzano FO, Del-Aguila-Arcentales S, et al. Prescription, over-the-counter (OTC), herbal, and other treatments and preventive uses for COVID-19. Environmental and Health Management of Novel Coronavirus Disease (COVID-19). 2021. pp. 379–416.

27. Moher D, Shamseer L, Clarke M, Ghersi D, Liberati A, Petticrew M, et al. Preferred reporting items for systematic review and meta-analysis protocols (prisma-p) 2015 statement. Syst Rev. 2015;4: 1.

28. Herzog R, Álvarez-Pasquin MJ, Díaz C, Del Barrio JL, Estrada JM, Gil Á. Are healthcare workers intentions to vaccinate related to their knowledge, beliefs and attitudes? A systematic review. BMC Public Health. 2013;13: 154.

29. Sadio A, Gbeasor-Komlanvi F, KONU R, Bakoubayi A, Tchankoni M, Bitty-Anderson A, et al. Assessment of self-medication practices in the context of Covid-19 outbreak in Togo. BMC Public Health. 2021;21: 58.

30. Quispe-Cañari JF, Fidel-Rosales E, Manrique D, Mascaró-Zan J, Huamán-Castillón KM, Chamorro–Espinoza SE, et al. Self-medication practices during the COVID-19 pandemic among the adult population in Peru: A cross-sectional survey. Saudi Pharm J. 2021;29: 1–11.

31. Zavala-Flores E, Salcedo-Matienzo J. Medicación prehospitalaria en pacientes hospitalizados por COVID-19 en un hospital público de Lima-Perú. Acta Medica Peru. 2020;37: 393–395.

32. Miñan-Tapia A, Conde-Escobar A, Calderon-Arce D, Cáceres-Olazo D, Peña-Rios AJ, Carlos Donoso-Romero R. Factores asociados a la automedicación con fármacos relacionados a COVID-19 en estudiantes de ciencias de la salud de una ciudad peruana. SciELO Prepr. 2020; Not peer reviewed.

33. Nasir M, Chowdhury ASMS, Zahan T. Self-medication during COVID-19 outbreak: a cross sectional online survey in Dhaka city. Int J Basic Clin Pharmacol. 2020;9: 1325–1330.

34. Wegbom AI, Edet CK, Raimi O, Fagbamigbe AF, Kiri VA. Self-Medication Practices and Associated Factors in the Prevention and/or Treatment of COVID-19 Virus: A Population-

Based Survey in Nigeria. Front Public Heal. 2021;9: 606801.

35. Dare S, Eze E, Isaac E, Usman I, Ssempijja F, Bukenya E, et al. COVID-19 pandemic and behavioural response to self-medication practice in Western Uganda. medRxiv. 2021; Not peer reviewed.

36. Mansuri FMA, Zalat MM, Khan AA, Alsaedi EQ, Ibrahim HM. Estimating the public response to mitigation measures and self-perceived behaviours towards the COVID-19 pandemic. J Taibah Univ Med Sci. 2020;15: 278–283.

37. Bennadi D. Self-medication: A current challenge. J Basic Clin Pharm. 2014;5: 19.

38. Leelavanich D, Adjimatera N, Broese Van Groenou L, Anantachoti P. Prescription and non-prescription drug classification systems across countries: Lessons learned for thailand. Risk Manag Healthc Policy. 2020;13: 2753–2768.

39. Morgan DJ, Okeke IN, Laxminarayan R, Perencevich EN, Weisenberg S. Non-prescription antimicrobial use worldwide: A systematic review. Lancet Infect Dis. 2011;11: 692–701.

40. Torres NF, Chibi B, Middleton LE, Solomon VP, Mashamba-Thompson TP. Evidence of factors influencing self-medication with antibiotics in low and middle-income countries: a systematic scoping review. Public Health. 2019;168: 92–101.

41. World Health Organization. Therapeutics and COVID-19: living guideline. 2021 [cited 15 May 2021]. Available: https://www.who.int/publications/i/item/WHO-2019-nCoV-therapeutics-2021.1

42. Infectious Diseases Society of America (IDSA). IDSA Guidelines on the Treatment and Management of Patients with COVID-19. 2021 [cited 15 May 2021]. Available: https://www.idsociety.org/practice-guideline/covid-19-guideline-treatment-and-management/

43. Joint Statement by the International Pharmaceutical Federation and The World Self-Medication Industry. Responsible Self-Medication. 1999 [cited 20 Apr 2021]. Available: https://www.fip.org/file/1484

44. World Medical Association. WMA Statement on self-medication. 2017 [cited 10 Apr 2021]. Available: https://www.wma.net/policies-post/wma-statement-on-self-medication/

45. Limaye D, Limaye V, Krause G, Fortwengel G. A systematic review of the literature on survey questionnaires to assess self-medication practices. Int J Community Med Public Heal. 2017;4: 2620–2631.

46. Stull DE, Leidy NK, Parasuraman B, Chassany O. Optimal recall periods for patient-reported outcomes: Challenges and potential solutions. Curr Med Res Opin. 2009;25: 929–942.

47. Choi B, Granero R, Pak A. Catalog of bias in health questionnaires. Rev Costarr Salud Pública. 2010;19: 106–118.

48. Andrade C. The Limitations of Online Surveys. Indian J Psychol Med. 2020;42: 575–576.

Supporting information captions

S1 Checklist. PRISMA 2009 checklist.

S1 Table. Search strategies.

S2 Table. List of articles excluded after full-text review.

Figure 1

Click here to access/download;Figure;Figure.JPG

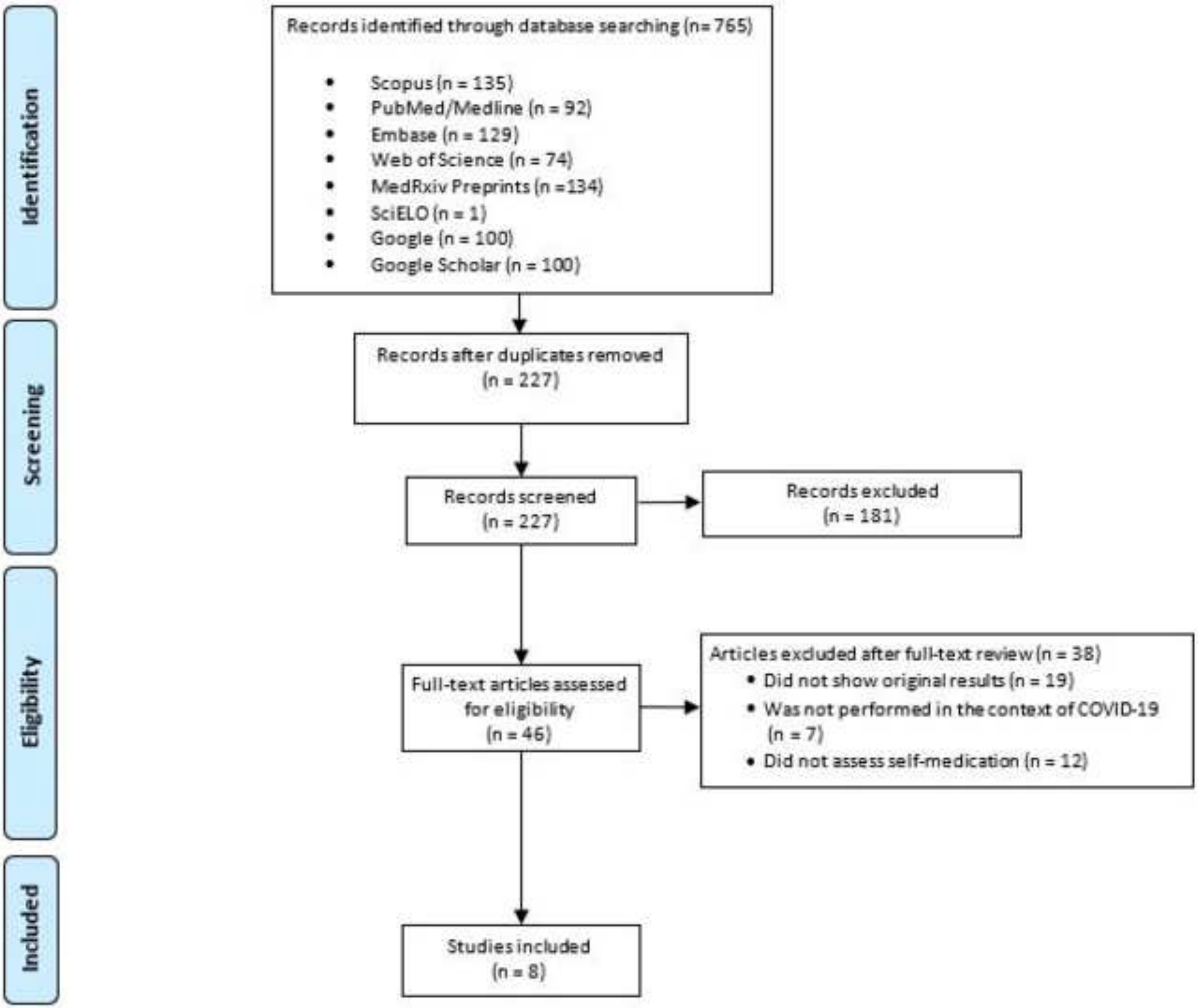

S1 Table

Click here to access/download
Supporting Information
S1 Table. Search strategies..docx

Click here to access/download
Supporting Information
S1 Checklist. PRISMA 2009 checklist..doc

S2 Table

Click here to access/download
Supporting Information
S2 Table. List of articles excluded after full-text review..docx

Self-medication practices to prevent or manage COVID-19: a systematic review

Short title: Self-medication practices for COVID-19

Authors:

Alvaro Quincho-Lopez[1]

Christeam A. Benites-Ibarra[2]

Maryori M. Hilario-Gomez[1]

Renatta Quijano-Escate[3]

Alvaro Taype-Rondan[4]

Affiliations:

[1]Universidad Nacional Mayor de San Marcos, Sociedad Científica de San Fernando, Lima, Peru

[2]Sociedad Científica de Estudiantes de Medicina de la Universidad Nacional del Santa, Nuevo Chimbote, Ancash, Peru

[3]Universidad Nacional San Luis Gonzaga, Sociedad Científica de Estudiantes de Medicina de Ica, Ica, Peru

[4]Universidad San Ignacio de Loyola, Unidad de Investigación para la Generación y Síntesis de Evidencias en Salud, Lima, Peru

Funding:

This study was self-funded by the authors.

Corresponding author:

Alvaro Taype-Rondan

Universidad San Ignacio de Loyola, Unidad de Investigación para la Generación y Síntesis de Evidencias en Salud

Av. La Fontana 750, Lima, Peru

alvaro.taype.r@gmail.com

Abstract

Background: Previous studies have assessed the prevalence and characteristics of self-medication in COVID-19. However, no systematic review has summarized their findings.

Objective: We conducted a systematic review to assess the prevalence of self-medication to prevent or manage COVID-19.

Methods: We used different keywords and searched studies published in PubMed, Scopus, Web of Science, Embase, two preprint repositories, Google, and Google Scholar. We included studies that reported original data and assessed self-medication to prevent or manage COVID-19. The risk of bias was assessed using the Newcastle–Ottawa Scale (NOS) modified for cross-sectional studies.

Results: We identified eight studies, all studies were cross-sectional, and only one detailed the question used to assess self-medication. The recall period was heterogeneous across studies. Of the eight studies, seven assessed self-medication without focusing on a specific symptom: four performed in the general population (self-medication prevalence ranged between <4% - 88.3%) and three in specific populations (range: 33.9% to 51.3%). In these seven studies, the most used medications varied widely, including antibiotics, chloroquine or hydroxychloroquine, acetaminophen, vitamins or supplements, ivermectin, and ibuprofen. The last study only assessed self-medication for fever due to COVID-19. Most studies had a risk of bias in the "representativeness of the sample" and "assessment of outcome" items of the NOS.

Conclusions: Studies that assessed self-medication for COVID-19 found heterogeneous results regarding self-medication prevalence and medications used. More well-designed and adequately reported studies are warranted to assess this topic.

Keywords: COVID-19, SARS-CoV-2, Self-Medication, Global Health, Pharmaceutical Preparations, Systematic Review

1 Introduction

With the progression of the COVID-19 pandemic, several medications have been proposed as potential candidates for this disease [1], most of which resulted in little or no benefit for the patients [2,3] or even in harms [4]. For example, hydroxychloroquine gained wide attention as a possible treatment for COVID-19 due to favorable results found in in-vitro or small uncontrolled studies [5]. However, later, randomized trials in hospitalized patients, such as the RECOVERY trial [6] and the Solidarity trial [3], failed to find any clinical benefit compared to usual care. This is similar to what happened with azithromycin [7–10], while there are still few well-designed trials that have assessed other medications such as ivermectin [11–13] or vitamins supplements [14,15].

Nonetheless, the fear of contracting the virus, low access to health services, and massive misinformation have led some people to self-medicate. According to the World Health Organization (WHO), self-medication "involves the use of medicinal products by the consumer to treat self-recognized disorders or symptoms." [16]. This may lead to unintended consequences, such as adverse events, unnecessary expenses, delay in attending professional evaluation, masking of symptoms, and drug interactions [17–19]. Self-medication prevalence varies according to several factors, such as the methodology used to assess self-medication [20], the population characteristics [20–22], and across different countries and contexts [23–25].

Previous studies have assessed the prevalence and characteristics of COVID-19 self-medication, reporting the use of several medications, herbal products, and dietary supplements as treatment or prevention for COVID-19 [26]. However, to date, no systematic review has summarized their findings.

Thus, in this systematic review, we aimed to assess the prevalence of self-medication for the prevention or management of COVID-19. In addition, we assessed the type of medication used, reasons to practice self-medication, from where such medications were obtained, and adverse events due to its practice.

2 Methods

This systematic review was reported according to the Preferred Reporting Items for Systematic Reviews and Meta-Analyses (PRISMA) guidelines (Supplementary Table S3) [27]. The study protocol has been registered at PROSPERO (CRD42021236191).

2.1 Data sources

Search strategies were constructed a priori using different terms related to 'COVID-19' and 'self-medication' (Supplementary Table S1). On February 4th, 2021, we searched PubMed, Scopus, Web of Science, Embase, two preprint repositories (MedRxiv and SciELO Preprints), and grey literature sources (Google and Google Scholar). No language restriction was applied. We planned to hire a translation service if any study written in a language other than English or Spanish was found. The search was restricted to include documents published in 2020 or 2021.

2.2 Inclusion criteria

We included original studies, either published in scientific journals or with full text available in preprint repositories, which reported original data and assessed the use of self-medication to prevent or manage COVID-19 as a primary or secondary outcome, in participants of any age and from any location.

We considered that a study assessed the use of self-medication in any of the following situations: 1) the study reported explicitly that self-medication was assessed (regardless of the definition used), or 2) the study assessed the use of medications without medical prescription.

2.3 Study selection

Two authors (AQL and MHG) independently selected the studies. For this, they first screened the search results by title and abstract according to the inclusion criteria using the web-based tool Rayyan (http://rayyan.qcri.org). Later, they reviewed the full text of the relevant studies to determine whether they were appropriate for study inclusion. Discrepancies were consulted with another author (ATR) and resolved by consensus.

2.4 Data extraction

Two authors (CBI and RQE) independently extracted the relevant data for the review using a pre-piloted Microsoft Excel spreadsheet. Again, any discrepancies were discussed with another author (ATR) and resolved with consensus.

The following variables were assessed for each study: author, year of publication, country, study design, setting, population, sample technique, date of data collection, number of participants, age, sex, how was self-medication assessed, the prevalence of self-medication, reasons for practicing self-medication, who recommended such medication, from where was the medication obtained, and adverse events related to its use.

2.5 Risk of bias assessment

Two authors (CBI and RQE) independently evaluated the risk of bias for each included study using the Newcastle–Ottawa Scale (NOS) adapted for descriptive cross-sectional studies [28]. This scale comprises two criteria: the selection criteria (representativeness of the sample, sample size, and nonresponders) and the outcome criteria (outcome assessment and statistical tests). Any discrepancy was discussed with another author (ATR).

2.6 Statistical analysis

Because studies were performed in different populations and using different definitions of self-medication, we did not perform meta-analyses and decided to present the results of each study separately.

3 Results

3.1 Study selection

A total of 765 records were found in the database search. After the duplicates were eliminated, 227 records were screened, of which 46 documents were assessed in full-text. Lastly, 38 papers were

excluded (which are detailed in Supplementary Table S2), and 8 papers were included, all of which were cross-sectional studies (Fig. 1).

[Insert Figure 1]

Fig 1. Flow diagram of study selection.

3.2 Methodological quality of the included studies

We assessed the risk of bias using the NOS. The study performed by Sadio [29] included two samples: one obtained using a probabilistic sample, and the other used a convenience sample. Thus, we assessed the risk of bias of these two samples separately.

Of the eight studies, only one (Sadio probabilistic sample) fulfilled the five stars in the NOS scale. In addition, the Sadio non-probabilistic sample, along with the other three studies, received four out of five stars. All studies except one (Sadio probabilistic sample) failed in representing the sample item of the selection criteria (Table 1).

Table 1. Risk of bias assessment of included studies using the Newcastle–Ottawa Scale adapted for cross-sectional studies.

Study: Author (year)	Final score	Representativeness of the sample[1]	Sample size[2]	Not respondents[3]	Outcome evaluation[4]	Statistical test[5]
Dare (2021)	2/5			★		★
Nasir (2020)	1/5				★	
Quispe-Cañari (2021)	4/5		★	★	★	★
Wegbom (2021)	3/5		★	★		★
Miñan-Tapia (2020)	4/5		★	★	★	★
Sadio (2021)[6]	5/5	★	★	★	★	★
Sadio (2021)[7]	4/5		★	★	★	★
Zavala-Flores (2020)	1/5				★	
Mansuri (2020)	4/5		★	★	★	★

[1]Representativeness of the sample: One star was given to studies with random sampling or census.
[2]Sample size: One star was given to studies with justified and satisfactory sample size.
[3]Non-respondents: If comparability between respondents' and nonrespondents' characteristics was established and the response rate was satisfactory, one star was given.
[4]Outcome evaluation: If the study explicitly mentioned how self-medication was defined and how long the recall period was, one star was given.
[5]Statistical test: One star was given if it did not use a complex sample and the sample had been calculated correctly, or if it used a complex sample and such sampling was considered to estimate the self-medication prevalence.
[6]Probability sampling.
[7]Nonprobability sampling.

3.3 Studies characteristics

Studies characteristics are shown in Table 2. The eight included studies reported data on self-medication practices in six different countries: three from Peru [30–32], one from Bangladesh [33], one from Togo [29], one from Nigeria [34], one from Uganda [35], and one from the Kingdom of Saudi Arabia [36]. All studies were cross-sectional. Regarding their publication, five studies were published as original reports [29,30,33,34,36], one was a letter to the editor [31], and two were preprints reporting original data [32,35]. Studies were performed from March to August 2020.

Table 2. Characteristics and findings of studies that assessed self-medication to prevent or manage COVID-19.

Study: Author (year)	Country (city) / date of study	Subjects (age, sex)	Survey application and self-medication definition	Prevalence of self-medication for COVID-19 prevention or management (overall and most-used medications)
**Studies that assessed self-medication without focusing on a specific symptom, performed in the general population:**				
Dare (2021) - preprint	Uganda (Western region) / from June to August 2020	Adults from Uganda western cities (age: 43.8% between 25 and 34 years, female: 45.2%), N = 272	• In-person and online survey (convenience sampling).<br>• Self-medication assessment was not detailed.<br>• Recall period: during the COVID-19 pandemic.	• **Overall:** 57%.
Nasir (2020)	Bangladesh (Dhaka city) / from April to June 2020	Adults (age: 50.5% between 45 and 54 years, female: 55.3%), N = 626	• Open online survey (convenience sampling).<br>• Self-medication was defined as taking medications without prescription.<br>• Recall period: during the COVID-19 outbreak.	• **Overall:** 88.3%.<br>• Ivermectin (77.2%), azithromycin (54.2%), montelukast (43.1%), calcium supplements (41.4%), doxycycline (40.3%), hydroxychloroquine (20.4%).
Quispe-Cañari (2021)	Peru / June 2020	Adults (median age: 23 years, female: 54.5%), N = 3792	• Open online survey (convenience sampling).<br>• Respondents were asked to indicate whether they consumed any of the following medications: acetaminophen, ibuprofen, azithromycin, hydroxychloroquine, penicillin, or antiretrovirals.<br>• Recall period: during the COVID-19 lockdown.	• **Overall:** not reported, but taking into account the prevalence for each medication, overall prevalence was < 4%.<br>• **For prevention:** azithromycin (0.2%), acetaminophen (0.2%), hydroxychloroquine (0.1%), antiretrovirals (0.1%), penicillin (0.1%) ibuprofen (0.03%).<br>• **For management:** acetaminophen (1.7%), azithromycin (0.6%), ibuprofen (0.2%), antiretrovirals (0.1%), hydroxychloroquine (0.03%), penicillin (0.03%).
Wegbom (2021)	Nigeria / from June to July 2020	Adults (mean age: 42.2 years, female: 57.1%), N = 461	• Open online survey (convenience sampling).<br>• Self-medication was defined as taking medications for the prevention or management of COVID-19 without prescription by medically qualified personnel.<br>• Recall period: not detailed.	• **Overall:** 41%.<br>• Vitamin C or multivitamin (21.3%), antimalarial drugs other than hydroxychloroquine and chloroquine (19.3%), amoxicillin (10.2%), ciprofloxacin (6.1%), herbal products (4.1%), metronidazole (3.5%), erythromycin (2.2%), hydroxychloroquine or chloroquine (1.3%).
**Studies that assessed self-medication without focusing on a specific symptom, performed in a specific population:**				
Miñan-Tapia (2020) - preprint	Peru (Tacna) / from June to July 2020	Undergraduate students of health-related careers from two universities (median age: 21 years, female: 69.1%), N = 718	• Online, convenience sampling.<br>• Self-medication was defined as using any of the pre-specified 14 medicines for COVID-19 without medical prescription.<br>• Recall period: during the past three months.	• **Overall:** 51.3%<br>• Acetaminophen (21.2%), ibuprofen (10.8%), dexamethasone (5.5%), aspirin (4.4%), azithromycin (2.5%).

Sadio (2021)	Togo (Lomé) / from April to May 2020	Adults from any of these five sectors: health care, air transport, police, road transport, and informal (median age: 36 years, female: 28.4%), N = 955	• In-person survey through recruitment, open invitation, and random sampling (two or three stages).<br>• Self-medication was defined as the use of drugs alleged to treat or prevent COVID-19 without a physician order.<br>• Recall period: 2 weeks.	• **Overall:** 34.2%<br>• Vitamin C (27.6%), traditional medicine (10.2%), chloroquine/hydroxychloroquine (2.0%), azithromycin (1.2%).
Zavala-Flores (2020) – letter to the editor	Peru (Lima) / June 2020	Hospitalized adults with COVID-19 (Mean age: 60.3 years, Female: 30.3%), N = 132	• The authors included all patients hospitalized during a specific week.<br>• Self-medication was defined as using some COVID-19 medication before hospitalization without medical prescription.<br>• Recall period: since the COVID-19 diagnosis.	• **Overall (for COVID-19 management):** 33.9%<br>• Antibiotics (28.3%), ivermectin (20.7%), corticosteroids (17.0%), acetaminophen (12.3%), aspirin (4.7%), NSAID 84.7%), hydroxychloroquine (0.9%), others (1.9%).
**Studies that assessed the use of medications for a specific symptom:**				
Mansuri (2020)	Kingdom of Saudi Arabia / from March to April 2020	Adults (age: 60.8% were less than 40 years, female: 60.3%), N = 388	• An online electronic survey, convenience sampling.<br>• Self-medication was assessed using the question, "Are you self-medicating for fever?"<br>• Recall period: during the COVID-19 pandemic.	• **Overall:** 35.1% to manage fever.

NSAID, nonsteroidal anti-inflammatory drug

Regarding the population, five studies were conducted targeting the general population, one was conducted in hospitalized patients with COVID-19 [31], one in healthcare undergraduate students [32], and one in workers from five sectors (health care, air transport, police, road transport, and informal) [29].

The eight studies included between 132 and 3,792 participants. Five studies mentioned the summary measure (mean or median) of the participants' age. Such summary measure (either mean or median) ranged from 21 to 60.3 years. All studies were performed in adults. The percentage of females ranged from 28.4% to 69.1% (Table 2).

3.4 Definition of self-medication

All studies assessed self-medication through self-report. The questions used to assess self-medication were explicitly mentioned only in the one study [36], while other five studies report having asked for the use of medicine without prescription [29,31–34], and two studies gave no details regarding how self-medication was measured in their surveys [30,35]. Moreover, three of the eight studies [30,32,36] reported collecting only a pre-specified list of medications.

Regarding the recall period for which self-medication was assessed: three studies assessed it during the COVID-19 pandemic [33,35,36], one during the COVID-19 lockdown [30], one during the past three months [32], one during the past two weeks [29], one since the COVID-19 diagnosis [31], and one did not detail the recall period [34] (Table 2).

3.5 Prevalence of self-medication and most frequently used medications

As showed in Table 2, We classified the eight included studies into three groups: 1) those that assessed self-medication for COVID-19 prevention or management without focusing on a specific symptom, and 2) those that assessed self-medication for a specific COVID-19 symptom.

Seven studies were included in the first group (assessed self-medication for COVID-19 prevention or management without focusing on a specific symptom). Of these, four were performed in the general population (with a range of self-medication prevalence between <4% and 88.3%) and three in specific populations (which reported a self-medication prevalence of 33.9% for hospitalized adults with COVID-19, 34.2% in adults who worked in five assessed sectors, and 51.3% in undergraduate students of health-related careers).

Of these seven studies, six mentioned the list of medications reported by the participants. The reported medications were different across the studies: six studies reported the consumption of antibiotics, five of chloroquine or hydroxychloroquine, three of acetaminophen, three of vitamins or supplements, two of ivermectin, and two of ibuprofen.

In addition, consumption preferences varied across studies. For example, when medications were sorted in the order of highest to lowest consumption, vitamins/supplements occupied the first place in two studies, but the fourth place in another study (Table 2).

The second group (studies that assessed the use of medication for a specific symptom) included one study performed on the general population of Saudi Arabia, which found that 35.1% of the surveyed individuals used self-medication for fever [36] (Table 2).

3.6 Reasons to practice self-medication, from where were the medications obtained, and adverse effects

Four of the eight included studies mentioned the reasons for self-medication. However, each study assessed the reasons differently. Thus, one study referred only to the symptoms that motivated participants to self-medicate, and the other three collected a plethora of reasons, including the fear of stigmatization, fear of quarantine, affordability, the convenience of self-medication, and patients believing that the symptoms were not severe (Table 3).

Only one study [34] specified the source of the patients' self-medications. This study was performed on adults from Nigeria. The most common sources reported were the pharmacy (73.9%) and the patent medicine vendor (23.6%).

Only two studies reported whether adverse events occurred in those who self-medicate. One study performed on Undergraduate students of health-related careers found that 11.7% of those who self-medicate presented with side effects of self-medication in the past three months [32]. Nonetheless, this study did not specify the adverse effects that occurred. The second study, performed in adults from Nigeria, reported body rash (23.1%) followed by worsened condition (17.3%), yellowish eyes (7.7%), swollen face (3.8%), and vomiting of blood and severe diarrhea (5.8%) [34].

Table 3. Reasons to practice self-medication, from where were the medications obtained, and adverse effects

Study: Author (year)	Reasons to practice self-medication	Obtained from	Adverse effects
Nasir (2020)	Fever (37.6%), throat pain (28.8%), dry cough (14.2%), loss of smell (9.2%), loss of taste (3.5%), body ache (5.0%), and rarely diarrhea (1.7%)	NR	NR
Wegbom (2021)	Fear of stigmatization or discrimination (79.5%), fear of quarantine or self-isolation (77.3%), fear of infection or contact with a suspected or known COVID-19 patient (76.3%), delay in receiving treatment at health facilities" (55.6%), influence of friends" (55.2%), social media (54.3%), nonavailability of medications for COVID-19 treatment in the health facilities (53%), emergency illness (49.1%), delay in getting hospital services (28.1%), distance to the health facility (23%), proximity of the pharmacy (21%), nonavailability of medicine in a health facility (19.3%), and health facility charges (15.3%)	Pharmacy (73.9%), patent medicine vendor (23.6%), hospital (7.6%), hawkers (4.5%), faith-based outlets, and herbalists (2.1%, each)	Body rash (23.1%) followed by worsened condition (17.3%), yellowish eyes (7.7%), swollen face (3.8%), and vomiting of blood and severe diarrhea (5.8%).
Dare (2021)	Self-medication is affordable (37%) and convenient (32%), lack the means to get to the health	NR	NR

	facility/hospital (15%), fear of being diagnosed COVID-19 positive (9%), fear of visiting health facility or hospital (7%)		
Miñan-Tapia (2020)	Believing that the symptoms were not severe enough to go to a doctor (64.3%), referring to family/friends that are nonmedical health professionals (34.9%), and owing to economic reasons or use of over-the-counter medications (34.9%)	NR	11.7% participants reported adverse events (not detailed)

NR: not reported

Note: The percentage was only based on the people who self-medicated in each study.

4 Discussion

To our knowledge, this is the first systematic review to assess the self-medication prevalence for COVID-19 prevention or management. It assessed the most important databases and sources for grey literature search and preprints repositories; without language limitations. Thus, our results should reflect the state of knowledge until the search date.

Self-medication is a global phenomenon that may involve health risks at both the individual and community levels [19,37]. Previous studies have found that self-medication is a common practice. Three systematic reviews conducted in Iran [23], Ethiopia [25], and India [24]; reported a prevalence of 53%, 44%, and 53.6%, respectively; for a recall period that ranged from a single day to 6 months.

In our review, we only found eight studies. Although the included studies were performed in six countries, the lack of studies in some regions such as North America, the Middle East, North Africa, and Oceania is evident. It is expected that the intercountry differences in drug promotion, regulations, and the possibility of accessing some medications without a prescription can influence the self-medication patterns [38,39]. Moreover, seven of the eight included studies were performed in low- and middle-income countries. While self-medication may be higher in such countries, due to the conditions and structure of the health system [40], it is not possible to make solid comparisons due to the heterogeneity in the self-medication assessment across studies.

Some of the included studies assessed over-the-counter medications such as acetaminophen or nonsteroidal anti-inflammatory drugs, which are used for the symptom management of COVID-19, along with several other diseases. However, the included studies have also found an heterogeneous

prevalence of self-medication with medications that have not proven to benefit in the prevention or management of COVID-19 (such as antibiotics, chloroquine or hydroxychloroquine, vitamins or supplements, ivermectin, and antiasthmatics), as stated by the current COVID-19 guidelines by WHO and IDSA [41,42].

In fact, the adverse effects of some of these medications are of great concern, such as antibiotic resistance due to extensive antibiotic use, bleeding caused by aspirin use, inhibition of the immune system caused by corticosteroids, or arrhythmia caused by hydroxychloroquine [4]. Some healthcare organizations have issued statements on self-medication. For instance, the WHO recognizes that a "successful" (i.e., beneficial) self-medication can be achieved in many countries only by improving individuals' knowledge and education level in such a way that the potential damages of this practice can be avoided [16]. Similarly, the International Pharmaceutical Federation, in tandem with the World Self-Medication Industry [43], and the World Medical Association [44], emphasizes the responsible use of non-prescription medications.

Similar to that found in previous systematic reviews [20,21], the included studies in our review tended to use different questions to assess self-medication, or they did not specify the question used. Moreover, studies have established different recall periods, and even those that assessed self-medication since the beginning of the pandemic would have different time frames depending on the month in which they were performed. This lack of clear information prevents direct comparisons between studies and the subsequent meta-analysis of their results [45], adding a potential source of bias that affects the internal validity of the results [45,46]. In addition, it is possible that studies that evaluated self-medication through a pre-specified list of medications reported biased prevalences, since these medications may not have included all the most commonly used medications [47].

The included studies had an important risk of bias, mainly in the "representativeness of the sample" domain of the NOS, the worst-rated item in the risk of bias assessment. This prevents the adequate extrapolation of the results [48], which should be taken into account when interpreting the results of this review.

Due to the limitations of the primary studies included in this review, more well-designed and adequately reported studies are needed, as well as the use of standardized self-medication definitions across studies. Moreover, since the COVID-19 status is changing rapidly, future studies are required to assess if there is any variation in self-medication trends between COVID-19 waves and after introducing the massive vaccination.

The findings of this review suggest that there is an important though heterogeneous prevalence of self-medication to prevent or manage COVID-19. This includes some medications that have not shown any benefit so far and may therefore expose people to unnecessary adverse events. The results vary widely across studies, suggesting that each context (region, country) has its own self-medication patterns and impact, calling on local health authorities to promote research and interventions to reduce potential self-medication adverse consequences.

5 Conclusion

We identified eight studies that assessed the use of self-medication for the prevention or management of COVID-19. Only one detailed the question used to assess self-medication. The recall period was heterogeneous across studies. With respect to the seven studies that assessed self-medication without focusing on a specific symptom: five performed in the general population (self-medication prevalence ranged between <4% - 88.3%) and three in specific populations. In these studies, the most used medications varied widely. More well-designed and adequately reported studies are warranted in this regard.

Acknowledgments

None.

References

1. Sanders JM, Monogue ML, Jodlowski TZ, Cutrell JB. Pharmacologic Treatments for Coronavirus Disease 2019 (COVID-19): A Review. JAMA - J Am Med Assoc. 2020;323:

1824–1836.

2. Lam S, Lombardi A, Ouanounou A. COVID-19: A review of the proposed pharmacological treatments. Eur J Pharmacol. 2020;886: 173451.

3. WHO Solidarity Trial Consortium. Repurposed Antiviral Drugs for Covid-19 — Interim WHO Solidarity Trial Results. N Engl J Med. 2021;384: 497–511.

4. Kim MS, An MH, Kim WJ, Hwang TH. Comparative efficacy and safety of pharmacological interventions for the treatment of COVID-19: A systematic review and network meta-analysis. PLoS Med. 2020;17: 1–28.

5. Ng TSB, Leblanc K, Yeung DF, Tsang TSM. Medication use during COVID-19. Can Fam Physician. 2021;67: 171–179.

6. RECOVERY Collaborative Group, Horby P, Mafham M, Al. E. Effect of Hydroxychloroquine in Hospitalized Patients with Covid-19. N Engl J Med. 2020;383: 2030–2040.

7. Furtado R, Berwanger O, Fonseca H, Al. E. Azithromycin in addition to standard of care versus standard of care alone in the treatment of patients admitted to the hospital with severe COVID-19 in Brazil (COALITION II): a randomised clinical trial. Lancet. 2020;396: 959–967.

8. Hinks TSC, Cureton L, Knight R, Wang A, Cane JL, Barber VS, et al. Azithromycin versus standard care in patients with mild-to-moderate COVID-19 (ATOMIC2): an open-label, randomised trial. Lancet Respir Med. 2021;19: 1–11.

9. Oldenburg CE, Pinsky BA, Brogdon J, Chen C, Ruder K, Zhong L, et al. Effect of Oral Azithromycin vs Placebo on COVID-19 Symptoms in Outpatients with SARS-CoV-2 Infection: A Randomized Clinical Trial. JAMA - J Am Med Assoc. 2021;326: 490–498.

10. PRINCIPLE Trial Collaborative Group, Butler CC, Dorward J, Yu LM, Gbinigie O, Hayward G, et al. Azithromycin for community treatment of suspected COVID-19 in people at increased risk of an adverse clinical course in the UK (PRINCIPLE): a randomised, controlled, open-label, adaptive platform trial. Lancet. 2021;397: 1063–1074.

11. Bartoszko JJ, Siemieniuk RAC, Kum E, Qasim A, Zeraatkar D, Ge L, et al. Prophylaxis against covid-19: Living systematic review and network meta-analysis. BMJ. 2021;373: 1–12.

12. López-Medina E, López P, Hurtado IC, Dávalos DM, Ramirez O, Martínez E, et al. Effect of Ivermectin on Time to Resolution of Symptoms Among Adults With Mild COVID-19: A

Randomized Clinical Trial. Jama. 2021; 1–10. doi:10.1001/jama.2021.3071

13. Vallejos J, Zoni R, Bangher M, Villamandos S, Bobadilla A, Plano F, et al. Ivermectin to prevent hospitalizations in patients with COVID-19 (IVERCOR-COVID19) a randomized, double-blind, placebo-controlled trial. BMC Infect Dis. 2021;21: 635.

14. Thomas S, Patel D, Bittel B, Wolski K, Wang Q, Kumar A, et al. Effect of High-Dose Zinc and Ascorbic Acid Supplementation vs Usual Care on Symptom Length and Reduction among Ambulatory Patients with SARS-CoV-2 Infection: The COVID A to Z Randomized Clinical Trial. JAMA Netw Open. 2021;4: 2–11.

15. Murai IH, Fernandes AL, Sales LP, Pinto AJ, Goessler KF, Duran CSC, et al. Effect of a Single High Dose of Vitamin D3on Hospital Length of Stay in Patients with Moderate to Severe COVID-19: A Randomized Clinical Trial. JAMA - J Am Med Assoc. 2021;325: 1053–1060.

16. World Health Organization. Guidelines for the regulatory assessment of medicinal products for use in self-medication. 2000 [cited 10 Apr 2021]. Available: https://apps.who.int/iris/handle/10665/66154

17. Tasnim S, Hossain M, Mazumder H. Impact of rumors and misinformation on COVID-19 in Social Media. J Prev Med Public Heal. 2020;53: 171–174. doi:10.3961/JPMPH.20.094

18. Onchonga D, Omwoyo J, Nyamamba D. Assessing the prevalence of self-medication among healthcare workers before and during the 2019 SARS-CoV-2 (COVID-19) pandemic in Kenya. Saudi Pharm J. 2020;28: 1149–1154.

19. Hughes CM, Mcelnay JC, Fleming GF. The benefits and risks of self-medication. Drug Saf. 2001;14: 1027–1037.

20. Shehnaz SI, Agarwal AK, Khan N. A systematic review of self-medication practices among adolescents. J Adolesc Heal. 2014;55: 467–483.

21. Jerez-Roig J, Medeiros LFB, Silva VAB, Bezerra CLPAM, Cavalcante LAR, Piuvezam G, et al. Prevalence of Self-Medication and Associated Factors in an Elderly Population: A Systematic Review. Drugs and Aging. 2014;31: 883–896.

22. Behzadifar M, Behzadifar M, Aryankhesal A, Ravaghi H, Baradaran HR, Sajadi HS, et al. Prevalence of self-medication in university students: Systematic review and meta-analysis. East Mediterr Heal J. 2020;26: 846–857.

23. Azami-Aghdash S, Mohseni M, Etemadi M, Royani S, Moosavi A, Nakhaee M. Prevalence and cause of self-medication in Iran: A systematic review and meta-analysis article. Iran J

Public Health. 2015;44: 1580–1593.

24. Rashid M, Chhabra M, Kashyap A, Undela K, Gudi SK. Prevalence and Predictors of Self-Medication Practices in India: A Systematic Literature Review and Meta-Analysis. Curr Clin Pharmacol. 2019;15: 90–101.

25. Sisay M, Mengistu G, Edessa D. Epidemiology of self-medication in Ethiopia: A systematic review and meta-analysis of observational studies. BMC Pharmacol Toxicol. 2018;19: 56.

26. Yáñez JA, Chung SA, Román BR, Hernández-Yépez PJ, Garcia-Solorzano FO, Del-Aguila-Arcentales S, et al. Prescription, over-the-counter (OTC), herbal, and other treatments and preventive uses for COVID-19. Environmental and Health Management of Novel Coronavirus Disease (COVID-19). 2021. pp. 379–416.

27. Moher D, Shamseer L, Clarke M, Ghersi D, Liberati A, Petticrew M, et al. Preferred reporting items for systematic review and meta-analysis protocols (prisma-p) 2015 statement. Syst Rev. 2015;4: 1.

28. Herzog R, Álvarez-Pasquin MJ, Díaz C, Del Barrio JL, Estrada JM, Gil Á. Are healthcare workers intentions to vaccinate related to their knowledge, beliefs and attitudes? A systematic review. BMC Public Health. 2013;13: 154.

29. Sadio A, Gbeasor-Komlanvi F, KONU R, Bakoubayi A, Tchankoni M, Bitty-Anderson A, et al. Assessment of self-medication practices in the context of Covid-19 outbreak in Togo. BMC Public Health. 2021;21: 58.

30. Quispe-Cañari JF, Fidel-Rosales E, Manrique D, Mascaró-Zan J, Huamán-Castillón KM, Chamorro–Espinoza SE, et al. Self-medication practices during the COVID-19 pandemic among the adult population in Peru: A cross-sectional survey. Saudi Pharm J. 2021;29: 1–11.

31. Zavala-Flores E, Salcedo-Matienzo J. Medicación prehospitalaria en pacientes hospitalizados por COVID-19 en un hospital público de Lima-Perú. Acta Medica Peru. 2020;37: 393–395.

32. Miñan-Tapia A, Conde-Escobar A, Calderon-Arce D, Cáceres-Olazo D, Peña-Rios AJ, Carlos Donoso-Romero R. Factores asociados a la automedicación con fármacos relacionados a COVID-19 en estudiantes de ciencias de la salud de una ciudad peruana. SciELO Prepr. 2020; Not peer reviewed.

33. Nasir M, Chowdhury ASMS, Zahan T. Self-medication during COVID-19 outbreak: a cross sectional online survey in Dhaka city. Int J Basic Clin Pharmacol. 2020;9: 1325–1330.

34. Wegbom AI, Edet CK, Raimi O, Fagbamigbe AF, Kiri VA. Self-Medication Practices and Associated Factors in the Prevention and/or Treatment of COVID-19 Virus: A Population-

Based Survey in Nigeria. Front Public Heal. 2021;9: 606801.

35. Dare S, Eze E, Isaac E, Usman I, Ssempijja F, Bukenya E, et al. COVID-19 pandemic and behavioural response to self-medication practice in Western Uganda. medRxiv. 2021; Not peer reviewed.

36. Mansuri FMA, Zalat MM, Khan AA, Alsaedi EQ, Ibrahim HM. Estimating the public response to mitigation measures and self-perceived behaviours towards the COVID-19 pandemic. J Taibah Univ Med Sci. 2020;15: 278–283.

37. Bennadi D. Self-medication: A current challenge. J Basic Clin Pharm. 2014;5: 19.

38. Leelavanich D, Adjimatera N, Broese Van Groenou L, Anantachoti P. Prescription and non-prescription drug classification systems across countries: Lessons learned for thailand. Risk Manag Healthc Policy. 2020;13: 2753–2768.

39. Morgan DJ, Okeke IN, Laxminarayan R, Perencevich EN, Weisenberg S. Non-prescription antimicrobial use worldwide: A systematic review. Lancet Infect Dis. 2011;11: 692–701.

40. Torres NF, Chibi B, Middleton LE, Solomon VP, Mashamba-Thompson TP. Evidence of factors influencing self-medication with antibiotics in low and middle-income countries: a systematic scoping review. Public Health. 2019;168: 92–101.

41. World Health Organization. Therapeutics and COVID-19: living guideline. 2021 [cited 15 May 2021]. Available: https://www.who.int/publications/i/item/WHO-2019-nCoV-therapeutics-2021.1

42. Infectious Diseases Society of America (IDSA). IDSA Guidelines on the Treatment and Management of Patients with COVID-19. 2021 [cited 15 May 2021]. Available: https://www.idsociety.org/practice-guideline/covid-19-guideline-treatment-and-management/

43. Joint Statement by the International Pharmaceutical Federation and The World Self-Medication Industry. Responsible Self-Medication. 1999 [cited 20 Apr 2021]. Available: https://www.fip.org/file/1484

44. World Medical Association. WMA Statement on self-medication. 2017 [cited 10 Apr 2021]. Available: https://www.wma.net/policies-post/wma-statement-on-self-medication/

45. Limaye D, Limaye V, Krause G, Fortwengel G. A systematic review of the literature on survey questionnaires to assess self-medication practices. Int J Community Med Public Heal. 2017;4: 2620–2631.

46. Stull DE, Leidy NK, Parasuraman B, Chassany O. Optimal recall periods for patient-reported outcomes: Challenges and potential solutions. Curr Med Res Opin. 2009;25: 929–942.

47. Choi B, Granero R, Pak A. Catalog of bias in health questionnaires. Rev Costarr Salud Pública. 2010;19: 106–118.

48. Andrade C. The Limitations of Online Surveys. Indian J Psychol Med. 2020;42: 575–576.

Supporting information captions

S1 Checklist. PRISMA 2009 checklist.

S1 Table. Search strategies.

S2 Table. List of articles excluded after full-text review.

Dear editor and reviewers,

We have carefully considered the comments and have revised the application accordingly. We believe these comments will improve our manuscript. Our responses are presented below each of the reviewer's comments.

Reviewer #1

- R1C1: Needs an extensive language editing.
  - We agree with the recommendation, and a translator has checked the revised version of this manuscript.

- R1C2: Introduction doesn't give enough back-ground information about the topic. It should have included a background on burden of COVID 19, self-medication, rationale/justification to conduct this review, availability of previously published review article.
  - We agree with the recommendation; we added the following text in the second and third paragraphs of the introduction:
    - Line 36-40: "This may lead to unintended consequences, such as adverse events, unnecessary expenses, delay in attending professional evaluation, masking of symptoms, and drug interactions [17–19]. Self-medication prevalence varies according to several factors, such as the methodology used to assess self-medication [20], the population characteristics [20–22], and across different countries and contexts [23–25]."
    - Line 41-43: "Previous studies have assessed the prevalence and characteristics of COVID-19 self-medication, reporting the use of several medications, herbal products, and dietary supplements as treatment or prevention for COVID-19 [26]. However, to date, no systematic review has summarized their findings."

- R1C3: Line 36-37, is the objective of this review just assessing the frequency of self-medication for COVID-19? Many things are mentioned in the result section. Please include all the specific objectives of this review at the end of the introduction section.
  - We agree with the recommendation and added these items to the objective (end of the introduction), as following:
    - Line 44-46: "we aimed to assess the prevalence of self-medication for the prevention or management of COVID-19. In addition, we assessed the type of medication used, reasons to practice self-medication, from where such medications were obtained, and adverse events due to its practice."

- R1C4: Better if 'frequency' is replaced by 'prevalence' throughout the document.

- We agree with the recommendation and replaced "frequency" with "prevalence" throughout the manuscript.

- R1C5: Line 50-51, in the inclusion criteria you gave the definition of self-medication while you said the difference in definition is one of the limitations that hinder you from doing meta-analysis. so, remove the definition in the inclusion criteria. Ok to retain it in the introduction.
  - In order to clarify this, we have reformulated the text at the end of the "inclusion criteria" subheading of the methods, as follows:
    - Line 63-65: "We considered that a study assessed the use of self-medication in any of the following situations: 1) the study reported explicitly that self-medication was assessed (regardless of the definition used), or 2) study assessed the use of medications without medical prescription."

- R1C6: Include the following in the methods section, key words used for searching.
  - Thank you for your observation. The keywords used for the search in each database are available in the supplementary material (S1 Table). This is mentioned now in the second paragraph of the Methods:
    - Line 52-53: "Search strategies were constructed a priori using different terms related to 'COVID-19' and 'self-medication' (Supplementary Table S1)."

- R1C7: In your inclusion criteria nothing is said about the study participants in the retrieved studies (age (adult, children, or all), COVID status (positive, negative unknown)), region or geographic location where the studies were conducted, publication status (do you consider grey literature?).
  - We agree with the recommendation. We have added the following in the "inclusion criteria" subsection of the Methods:
    - Line 61-62: "in participants of any age and from any location."
  - Also, we have added the following in the "Data sources" subsection:
    - Line 54-55: "…and grey literature sources (Google and Google Scholar)."

- R1C8: 'Drug' and 'medication' are used interchangeably in the document, be consistent. I prefer 'medication' than 'drug' in your context.
  - We agree with the recommendation and replaced 'drug' by **'medication'** throughout the manuscript where appropriate.

- R1C9: No language restriction was applied. Do you get non-English articles? If so how or who did the translation? It should be mentioned in the methods section.
  - Thanks for the observation. In order to clarify this, we have added the following to the "Data sources" subsection of the Methods:

        - Line 55-56: "We planned to hire a translation service if any study written in a language other than English or Spanish was found."

Comments from the PDF file:

- R1C10: please avoid 'we' and express in passive form.
    - We agree. We rephrased in passive from the first paragraph of the Results, as follows:
        - Line 93-95: "227 records were screened, of which 46 documents were assessed in full-text. Lastly, 38 papers were excluded (which are detailed in Supplementary Table S2), and 8 papers were included"

- R1C11: is this mean or median.
    - Thanks for the observation. To clarify this, we have improved the text of the third paragraph of the "Study characteristics" subsection of the results, as follows:
        - Line 139-141: "Five studies mentioned the summary measure (mean or median) of the participants' age. Such summary measure (either mean or median) ranged from 21 to 60.3 years."

- R1C12: should this study (Quispe-Cañari et al.) included in this review? b/c it doesn't assess self-medication for COVID-19.
    - Thanks for the observation. However, the study of Quispe Cañari et al. assessed self-medication for COVID-19 prevention and management (table 2 of the results of the study, link: https://www.ncbi.nlm.nih.gov/pmc/articles/PMC7832015/). These are the results that we took into account.

- R1C13: is this for specific reason? no difference with studies done by Wegbom, sadio, ahmed...
    - We agree. We moved Zavala-Flores et al. to the group of studies that assessed self-medication without restrictions. Also, we merged tables 2 and 3.

- R1C14: if you expect that self-medication in LMIC is higher you have to first back it up with an evidence (reference). then you can mention however...
    - Thanks for the suggestion. To clarify this, in the third paragraph of the discussion now it says:
        - "While self-medication may be higher in such countries, due to the conditions and structure of the health system [40], it is not possible to make solid comparisons due to the heterogeneity in the self-medication assessment across studies."

        - 40.- Torres NF, Chibi B, Middleton LE, Solomon VP, Mashamba-Thompson TP. Evidence of factors influencing

self-medication with antibiotics in low and middle-income countries: a systematic scoping review. Public Health. 2019;168: 92–101. doi:10.1016/j.puhe.2018.11.018

- R1C15: inappropriate word choice (symptomatic)
  - We agree. We added the following (line 210-212): "Some of the included studies assessed **over-the-counter** medications such as acetaminophen or nonsteroidal anti-inflammatory drugs, which are **used for the symptom management of COVID-19**, along with several other diseases".

Reviewer #2

Introduction

- R2C1: Paragraph 1. The introduction part could be written with more information. For example, the authors may explain more on the examples of prevalence difference between countries, age group and occupation, in terms of the highest self-medication practices. A more thorough definition on self-medication would also be very useful, as this is the main focus of this paper, yet it is still unclear as it is only briefly mentioned in the introduction.
- R2C2: Paragraph 2. It would also be good if the authors described on the examples of drugs that has been proposed as the potential candidate for Covid-19, and the outcome of such treatments. The examples of direct and indirect consequences can also be further explained here, to make the introduction more informative.
- R2C3: Paragraph 3. Some description on the examples of medication used as self-treatment and indication in Covid would also be informative and will make the introduction more dynamic.
  - We agree with these 3 recommendations. Accordingly, we added the following text in the first paragraph of the introduction:
    - Line 25-32: "With the progression of the COVID-19 pandemic, several medications have been proposed as potential candidates for this disease [1], most of which resulted in little or no benefit for the patients [2,3] or even in harms [4]. For example, hydroxychloroquine gained wide attention as a possible treatment for COVID-19 due to favorable results found in in-vitro or small uncontrolled studies [5]. However, later, randomized trials in hospitalized patients, such as the RECOVERY trial [6] and the Solidarity trial [3], failed to find any clinical benefit compared to usual care. This is similar to what happened with azithromycin [7–10], while there are still few well-designed trials that have assessed other medications such as ivermectin [11–13] or vitamins supplements [14,15]."
  - Also, we added the following to the second and third paragraphs of the introduction:

        - Line 36-40: "This may lead to unintended consequences, such as adverse events, unnecessary expenses, delay in attending professional evaluation, masking of symptoms, and drug interactions [17–19]. Self-medication prevalence varies according to several factors, such as the methodology used to assess self-medication [20], the population characteristics [20–22], and across different countries and contexts [23–25]."
        - Line 41-43: "Previous studies have assessed the prevalence and characteristics of COVID-19 self-medication, reporting the use of several medications, herbal products, and dietary supplements as treatment or prevention for COVID-19 [27]. However, to date, no systematic review has summarized their findings."

Methods

- R2C4: Data source. It would be good if the authors can include the terms used for the papers search, as this is an important component for a systematic review.
    - Thank you for your observation. The keywords used for the search in each database are available in the supplementary material (S1 Table). This is mentioned now in the second paragraph of the Methods:
        - Line 52-53: "Search strategies were constructed a priori using different terms related to 'COVID-19' and 'self-medication' (Supplementary Table S1)."

Results

- R2C5: Definition of self-medication. If 9 of the 11 studies did not mention on the definition of self-medication, how did the author include these papers in the study? More explanation would be very useful. Please explain this in the manuscript. This showed the importance of providing the keywords for article search.
    - Thank you for your observation. To clarify this, we have added the following at the end of the first paragraph of the "inclusion criteria" subheading:
        - Line 63-65: "We considered that a study assessed the use of self-medication in any of the following situations: 1) the study reported explicitly that self-medication was assessed (regardless of the definition used), or 2) study assessed the use of medications without medical prescription."

- R2C6: Who suggested the practice of self-medication. In the results it stated that seeking advice from doctors and medical guideline are included as the source to the advice on self-medication. But if someone get an

advice from a doctor, would it still be defined as self-medication? This goes back to the concern on the definition of the self-medication and the selection of papers in this study. Please explain.

- Thanks for the observation. We have discussed the subject, and we consider that the self-medication definition is incompatible with the situations in which participants consulted with physicians regarding their medications.
- Accordingly, we have added this definition in the "inclusion criteria" subsection of the methods:
  - Line 63-65: "We considered that a study assessed the use of self-medication in any of the following situations: 1) the study reported explicitly that self-medication was assessed (regardless of the definition used), or 2) the study assessed the use of medications without medical prescription."
- Also, we reviewed again all the studies, and excluded 3 of them which did not match with this self-medication definition: Ahmed (2020), Kamarli Altun (2020), and Chauhan (2020). Accordingly, the review now includes only 8 studies, and all the articles have been updated.

Discussion

- R2C7: It would be good if the author may emphasize on the importance of the findings and why this paper is worth to be published.
  - We agree with the recommendation. Accordingly, we added the following as the last paragraph of the discussion (right before the conclusion):
    - Line 244-249: "The findings of this review suggest that there is an important though heterogeneous prevalence of self-medication to prevent or manage COVID-19. This includes some medications that have not shown any benefit so far and may therefore expose people to unnecessary adverse events. The results vary widely across studies, suggesting that each context (region, country) has its own self-medication patterns and impact, calling on local health authorities to promote research and interventions to reduce potential self-medication adverse consequences."

- R2C8: It would be good if the author may discuss on the future study or research gap related to the topic.
  - Thanks for the suggestion. We added the following as the penultimate paragraph of the discussion:
    - Line 239-243: "Due to the limitations of the primary studies included in this review, more well-designed and adequately reported studies are needed, as well as the use of standardized self-medication definitions across studies. Moreover, since the COVID-19 status is changing rapidly, future studies are required to assess if there is any variation in self-medication trends between COVID-19 waves and after introducing the massive vaccination."

Reviewer #3

- R3C1: Line 5 and 6 "Suppose the study aimed to assess the frequency of self-medication for the prophylaxis or management of COVID-19. In that case, I was wondering why the authors included studies that assessed self-medication for all reasons?"
    - We understand the concern of the reviewer. To clarify our inclusion criteria, we have changed the redaction as follows:
        - Line 59-62: “We included original studies, either published in scientific journals or with full text available in preprint repositories, which reported original data and assessed the use of self-medication to prevent or manage COVID-19 as a primary or secondary outcome, in participants of any age and from any location.”
        - Line 63-65: “We considered that a study assessed the use of self-medication in any of the following situations: 1) the study reported explicitly that self-medication was assessed (regardless of the definition used), or 2) the study assessed the use of medications without medical prescription."

- R3C2: Line 12 "The authors stated that they "identified 11 studies that assessed self-medication for the prevention or management of COVID-19." It is unclear how the authors linked the self-medication in the 7 studies that assessed "self-medication for any reason" to COVID-19. The authors' definition of self-medication did not specify COVID-19, e.g., Quispe-Cañari (2021). The respondents may be self-medicating for another ailment".
    - Thank you for your observation. To clarify this, we have added the following at the end of the first paragraph of the "inclusion criteria" subheading:
        - Line 63-65: "We considered that a study assessed the use of self-medication in any of the following situations: 1) the study reported explicitly that self-medication was assessed (regardless of the definition used), or 2) study assessed the use of medications without medical prescription. "
    - Regarding the study of Quispe Cañari et al, it assessed self-medication for COVID-19 prevention and management (table 2 of the results of the study, link: https://www.ncbi.nlm.nih.gov/pmc/articles/PMC7832015/). These are the results that we took into account.

- R3C3: Line 19 "It is unclear why the authors chose the term frequency instead of prevalence throughout the manuscript. 'Prevalence' may be more appropriate to use in this context".
    - We agree and have made such changes throughout the manuscript.

- R3C4: Line 29 "Replace 'now' with 'later'”

    - As we modified the writing of the manuscript, this suggestion is not valid anymore.

- R3C5: Line 33 to 35 “The sentence needs to be rephrased to reveal the intended meaning. Consider rephrasing to: “Previous studies have assessed the frequency and characteristics of COVID-19 self-medication to figure out which medications are being used that are ineffective or potentially dangerous and which factors predispose people to self-medication”
    - Thanks for the observation. We have changed the following:
        - Line 2-3: “Previous studies have assessed the prevalence and characteristics of self-medication in COVID-19. However, no systematic review has summarized their findings.”
        - Line 41-43: “Previous studies have assessed the prevalence and characteristics of COVID-19 self-medication, reporting the use of several medications, herbal products, and dietary supplements as treatment or prevention for COVID-19 [26]. However, to date, no systematic review has summarized their findings.”

- R3C6: Line 34 “The use of the term ‘useless’ is unsuitable for scientific writing. Consider using ‘ineffective’”
    - As we modified the writing of the manuscript, this suggestion is not valid anymore.

- R3C7: Line 43 “I wondered if it is possible to search all these databases in a single day without any pre-defined search guide. It will be good to state that the search terms were developed a priori for better clarity to any reader”.
    - We agree with the recommendation, So, in order to guide the readers, we added the following line in “Data sources” section:
        - Line 52-53: “Search strategies were constructed a priori using different terms related to ‘COVID-19’ and ‘self-medication’ (Supplementary Table S1).”

- R3C8: Line 44 to 45 “This is very nice. By not restricting, you avoid what is called “language bias”. However, since no language restriction was applied, it will be good for the authors to state how articles found that are not written in English were interpreted? Assuming none was found, the interpretation criteria should have been mentioned in the protocol, but unfortunately, it was not stated either in the protocol”.
    - Thanks for the observation. We added the following lines in the “Data sources” section to clarify this:
        - Line 55-56: “We planned to hire a translation service if any study written in a language other than English or Spanish was found.”

- R3C9: Line 120 “The Table title does not tally with the content. It states, “…. assessed self-medication of any drug for any reason”. The title will

assume that studies that assessed self-medication for the prevention and treatment of COVID-19 will not be included. The title needs to be rephrased to be all-encompassing. “Characteristics and findings of studies that assessed self-medication practices during the COVID-19 pandemic””

- We merged tables 2 y 3, and this new table 2 have the following title:
  - Line 131-132: “Characteristics and findings of studies that assessed self-medication to prevent or manage COVID-19.”

- R3C10: Table 3 “In the 3rd column in the 1st row, i.e., Mansuri (2020), it is unclear how you presented the Subjects (age, sex). It seems to be in percentage. The same applies to Zavala-Flores (2020). The unit of age should be stated, i.e., years, months, e.t.c.”
  - We agree and have made the change, in the first row of the third column of table 3 now it says “age: 60.8% **were less than** 40 **years**, female: 60.3%”. Then, in the last row of the third column of table 2, now it says, “Mean age: 60.3% **years**”.

- R3C11: Line 174 “Kindly rephrase to “Only one study [22] specified the source of the patients’ self-medications””
  - We agree and have made such change in the results. Please, see the second paragraph (line 180) of the “Reasons to practice self-medication, from where were the medications obtained, and adverse effects” subheading in the results.

- R3C12: Line 203 “Current guidelines??? It will be good to mention which guideline the authors are referring to. COVID-19 guideline?? By which body?? WHO, CDC etc”
  - We consider the recommendation, so we rephrased the sentence (4th paragraph of the discussion). Now it says:
    - Line 215-216: “As stated by the current COVID-19 guidelines by WHO and IDSA [41,42].”

- R3C13: Throughout the manuscript “The manuscript needs detailed proofreading and revision for its English”
  - We agree with the recommendation, and a translator has checked the revised version of this manuscript.

Reviewer #4

- R4C1: Page 3, paragraph 3: “…we aimed to assess the frequency of self-medication…”. Please explain the reason of using the term “frequency” rather than “prevalence”.
  - We have reviewed the differences between the two terms and agree with your suggestion. So that, we replaced ‘frequency’ by ‘prevalence’ throughout the manuscript.

- R4C2: Page 5-6, Table 1: Both the final score for Ahmed (2020) and Chauhan (2020) are zero. Please explain the rational of include these two articles in the systematic review although the scores are zero.

- Due to the commentaries of another reviewer, we have realized that 3 of the initially included studies did not fulfil the self-medication definition because they included medication taken due to a prescription. We have excluded these 3 studies from the results: Ahmed (2020), Kamarli Altun (2020), and Chauhan (2020).

- R4C3: Page 8, paragraph 2: "Of these seven studies, only two specified which question was asked, whereas the other five studies did not detail whether participants could report self-medication only for a prespecified list of drugs or whether it was an open question". The used of a prespecified list of drugs might narrow down the answers from the respondents. This subsequently might lead to biases in the study findings. This issue needs to be discussed in the discussion section.
  - We agree with your suggestion and decide to add the following sentence (6th paragraph of the discussion):
    - Line 232-234: "In addition, it is possible that studies that evaluated self-medication through a pre-specified list of medications reported biased prevalences, since these medications may not have included all the most commonly used medications [47]."

      47.- Choi B, Granero R, Pak A. Catalog of bias in health questionnaires. Rev Costarr Salud Pública. 2010;19: 106–118. Available: http://www.scielo.sa.cr/scielo.php?script=sci_arttext&pid=S1409-14292010000200008&lng=en.
