# Supplementary material for: Self-medication practices to prevent or manage COVID-19: A systematic review
Source: PLoS One. 2021 Nov 2;16(11):e0259317. doi: 10.1371/journal.pone.0259317 (PMC8562851; doi:10.1371/journal.pone.0259317)
Supplement: Attachment — Submitted filename: Response to Reviewers.docx [file pone.0259317.s007.docx]

Dear editor and reviewers,

We have carefully considered the comments and have revised the application accordingly. We believe these comments will improve our manuscript. Our responses are presented below each of the reviewer's comments.

Comments from reviewer #1:

R1C1: Line 55. I think you mean ''We had planned to hire a translation service if any study written in a language other than English have been found”. This is a sentence that should have been included in the protocol. but for the manuscript, better if replaced with 'but all the included articles were found written in English'.

- We agree with the recommendation and have changed that sentence according to your suggestion. Please, see line 55-56: “No language restriction was applied, **but all the included articles were found written in English or Spanish**”.

R1C2: Line 76: Add ‘data on’ before this sentence. Replace ‘assessed’ with ‘extracted’.

- We agree with the recommendation. Please, see line 76: “**Data on** the following variables were **extracted** for each study: author, year of publication, country…”.

R1C3: Line 106-107: Replace ‘representing’ with ‘fulfilling the representativeness of’. Replace ‘selection’ with ‘risk of bias assessment’.

- We agree with both recommendations. Please, see lines 106-107: “All studies except one (Sadio probabilistic sample) failed in **fulfilling the representativeness of** the sample item of the **risk of bias assessment** criteria (Table 1)”.

R1C4: Conclusions: All the objectives and main findings are not addressed in the conclusion.e.g. over all range of prevalence, the type of medication used, reasons to practice self-medication...

- We agree. We rephrased and added new sentences regarding the prevalence, type of medication, reasons, source, adverse events related to self-medication and quality of the included studies. Please, see lines 251-265: “Eight studies that assessed the use of self-medication for the prevention or management of COVID-19 **were identified**. The **definition and** recall period were heterogeneous across studies. **Of the eight studies, seven assessed self-medication without focusing on a specific symptom: four performed in the general population (self-medication prevalence ranged between <4% to 88.3%) and three in specific populations (range: 33.9% to 51.3%).** In these studies, the **most commonly** used medications varied widely, **including antibiotics, chloroquine or hydroxychloroquine, acetaminophen, vitamins or supplements, ivermectin, and ibuprofen. The last study only assessed self-medication for fever due to COVID-19.**

**Based on four studies, fever, fear of stigmatization, affordability of self-medication, and believing that the symptoms were not severe were the most common reasons to practice self-medication. The pharmacy was the preferred source of medications whereas body rash and worsened condition were the most frequent adverse event related to self-medication according to one and two studies, respectively. Almost all studies failed in fulfilling the representativeness of the sample item of the risk of bias assessment criteria. Thus,** more well-designed and adequately reported studies are warranted in this regard”.

R1C5: Line 255: Add ‘commonly’ after ‘most’.

- We agree with the recommendation. Please, see line 255: “In these studies, the **most commonly** used medications varied widely...”.
